# Photo-guided azopolymer hydrogel actuators

**DOI:** 10.1038/s41377-026-02411-5

**Published:** 2026-09-17

**Authors:** David Urban, Ryota Toyohara, Marcel Rey, Daniele Martella, Dag Roar Hjelme, Andrea Alessandrini, Toshiro Ohashi, Emiliano Descrovi

**Affiliations:** 1 Department of Electronic Systems, Norwegian University of Science and Technology, Trondheim, 7491 Norway; 2 Department of Applied Science and Technology, Politecnico di Torino, Torino, 10129 Italy; 3 Microsystems and Nanotechnology (MiNaLab), SINTEF Digital, Oslo, 0314 Norway; 4 Faculty of Engineering, Hokkaido University, Sapporo, 060-8628 Japan; 5 Institut für Physikalische Chemie, Universität Münster, Münster, 48149 Germany; 6 Dipartimento di Chimica “Ugo Schiff”, Università di Firenze, Sesto Fiorentino, 50019 Italy; 7 European Laboratory for Non-Linear Spectroscopy (LENS), Sesto Fiorentino, 50019 Italy; 8 Department of Physics, Informatics and Mathematics, University of Modena and Reggio Emilia, Modena, 41125 Italy; 9 CNR-Nanoscience Institute-S3, Modena, 41125 Italy

**Keywords:** Polymers, Optical manipulation and tweezers

## Abstract

Amorphous azopolymers are fascinating materials that can be deformed in arbitrary directions by light. However, they are so far used mostly for microfabrication, to anisotropically reshape dry polymer structures – essentially as a post-processing fabrication step. This is because the effect is known to be a plastic deformation, wherein the azopolymer is photo-softened and selectively reflows along the direction of the illumination polarization, owing to its polarization-dependent functional dyes. Crucially, such deformations are retained in the dark and cannot easily be overwritten by subsequent illumination. Once reflowed, there is no memory of the initial state, and sequential photo-deformations are added on top of each other. Consequently, to use the directional photo-deformation of amorphous azopolymers for dynamic and reconfigurable micro-actuators, e.g., in lab-on-chip applications, one would need to face this lack of overwritability, in addition to a lower deformability of larger structures, sticky behavior, and poor mechanical stability in water for some uses. Here, we show how azopolymer-hydrogel composites overcome these issues. By embedding azopolymer nanoparticles in hydrogel matrices, directional photo-deformation is ensured by the particles, while the compliant gel matrix neatly propagates deformations to the overall composite. Elastic restoring forces from the matrix also promote overwritability, such that microfabricated gel cubes display ample and directionally reconfigurable photo-deformations in water. Sequential illuminations with orthogonal linear polarizations produce alternating linear deformations up to twice the pristine cube side length, using illumination intervals down to five seconds. Finally, we introduce polarization-controlled, fully closeable microwells, with potential applications in biotechnology, microfluidics, and drug release.

## Introduction

The photo-deformation of amorphous side-chain azopolymers was reported by two groups in 1995, who observed the inscription of physical surface topographies, called gratings, on thin azopolymer films exposed to periodic light patterns^1,2^. Some years later, similar film structuring effects were observed to emerge from pure polarization patterns with uniform intensity illumination, suggesting directional, polarization-dependent deformation effects^3^. Eventually, the directional stretching of spherical azopolymer particles^4^ and pre-fabricated micro-posts^5^ along the polarization direction of visible light writing beams was directly evidenced. Thereafter, these materials have been more widely used in the realm of micro-and nanofabrication^6,7^, with new applications of the fundamental effect and its film surface structuring possibilities still emerging, e.g., in optics and photonics^8–10^. Simultaneously, these discoveries of intrinsic control over the directionality of azopolymer photo-deformation have also inspired a search for their utilization in dynamic settings, for example, as in vitro cell culture substrates with dynamically tunable topography^11–14^.

Yet, to envision azopolymers as dynamic actuators, whose shape can be directly, freely, and repeatedly controlled under a microscope, several challenges subsist. First, amorphous azopolymer deformations are plastic, and previous deformations cannot easily be overwritten since one usually has no means of reversing already inscribed deformations^15–17^. This limits continuous actuator control in the sense that actuators will accumulate light-induced deformations and, in practice, become increasingly deformed, along all the employed deformation directions combined. Secondly, a lack of mechanical stability under irradiation in an aqueous environment has been identified as a major challenge for biological or microfluidic applications^18,19^. Thirdly, while the photo-softening of azopolymers can facilitate properties such as self-healing of damaged films^20^, it also imparts stickiness to deforming microstructures. Consequently, adhesion between parts of the deforming structures and the underlying substrate seems to play a role in the much lower deformability of larger, supra-micrometric azopolymer structures^21,22^. Together, these factors have restricted the applications of this unique, albeit only partially understood, photo-mechanical phenomenon, which involves both statistical reorientation of azobenzene dyes under polarized light (Weigert effect)^23,24^ and photo-softening effects^25,26^.

On the other hand, stimuli-responsive hydrogels, i.e., externally or environmentally controlled water-swollen polymer networks, are a thriving field in material science, and many light-controlled photo-deformation approaches have been proposed in recent years^27,28^. At present, many of these approaches rely on local but substantially isotropic volume changes induced by, e.g., photothermal effects^29–31^ or photochemical reactions^32,33^. In some cases, these effects can produce directed actuator responses such as bending through mechanical constraints^34,35^. Intrinsic directionality of photo-mechanical responses can be achieved by combining swelling/deswelling and other actuation types with underlying material anisotropies, stemming, e.g., from aligned nanoplatelets^36,37^ or fiber structures^38^ in the gels, sometimes combined with composition gradients^39^ or additional, e.g., magnetic, stimuli^40^. However, in all of those approaches, the local photo-deformation direction is fixed during fabrication and may not be altered during use of the material.

Here, we introduce azopolymer-hydrogel (azoGel) composite actuators that embed azopolymer nanoparticles as photo-deformation drivers within hydrogel matrices. Interestingly, the composites inherit not only the particles’ capacity to be physically deformed in arbitrary directions – which we control via the illumination polarization – but also show good deformation overwritability ruled by physical particle-hydrogel interactions. In addition to quantifying these effects by deforming and tracking cubic microposts, we propose the use of these composites in printed microwells intended as light-controlled microvalves. We show how the latter can be opened and closed several times using only the illumination’s polarization as a signal.

## Results

### Polarization-directed photo-deformation of actuators

**Fig. 1:**
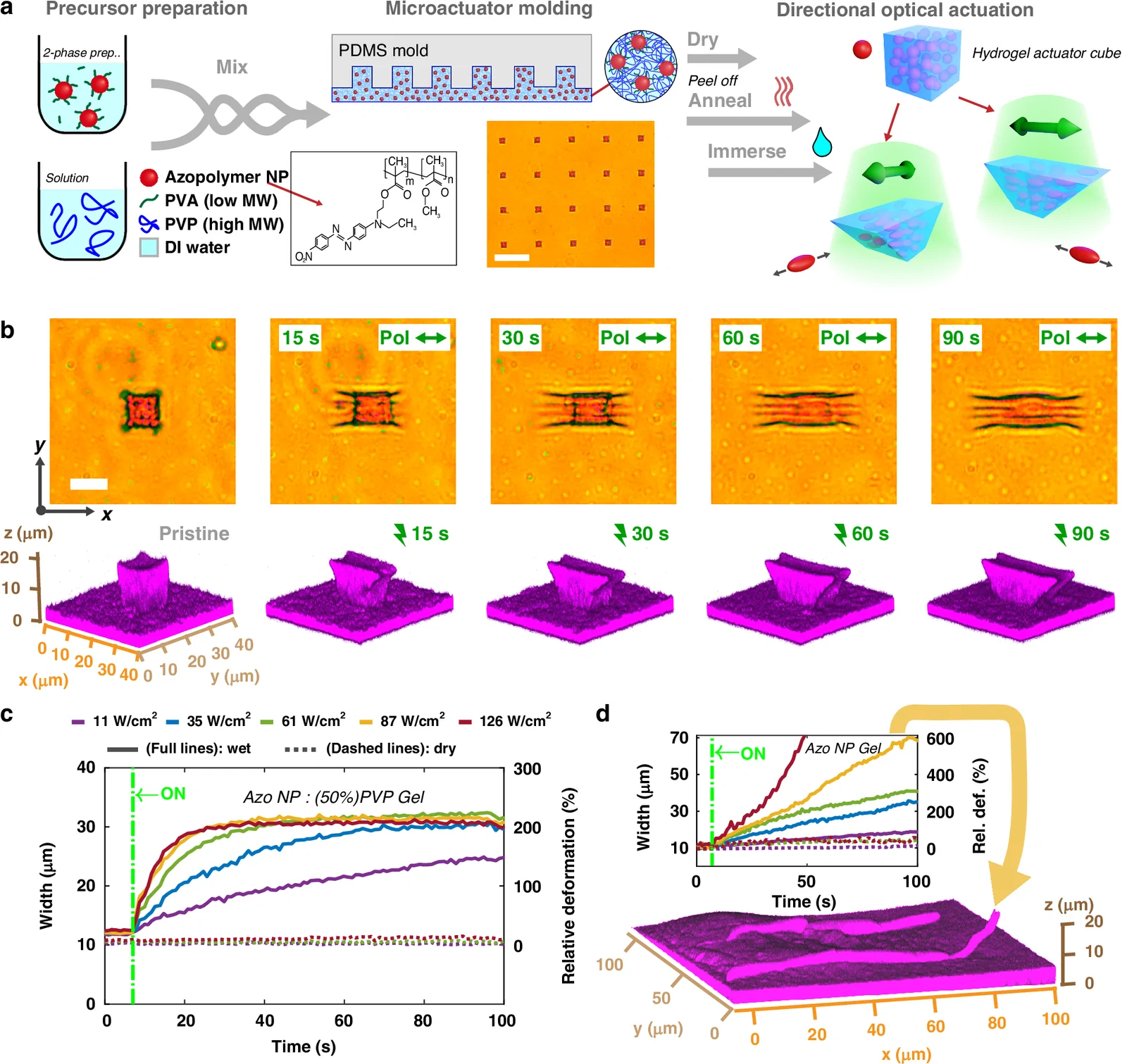
Directional photo-deformation of azoGel actuator cubes. **a** Schematic route for the fabrication of cubic micropost actuators. Left to right: precursor solutions including responsive azopolymer particles and water-soluble polymers (PVA/PVP), chemical structure of the azopolymer, molding procedure, optical microscope image of the resulting actuator array (scale bar, white: 50 μm), and schematic directional photo-deformation of cubes using polarized green laser illumination. **b** Optical microscopy (top row, scale bar: 10 μm) and 3D confocal microscopy reconstructions (bottom row) of micro-actuator cubes irradiated with linear *x*-oriented polarization for different time intervals at intensity *I* = 35 W cm^−2^. A residual layer from the molding procedure is visible. **c** Stretched width along the *x-*axis (polarization parallel) of actuator cubes vs. time at different irradiation intensities (see legend), using the same 50% PVP-containing actuators as in (**b**). Each line is based on the average deformation measured on four cubes (for readability, distribution bars are shown in Fig. S8). In swollen cubes, deformation saturates independently of illumination intensity. **d** Equivalent graphs acquired from cubes made of PVA-coated azopolymer particles only (i.e., without high molecular weight PVP). Deformation does not saturate in this case, and highly elongated wire-like shapes can be obtained (3D confocal image, bottom)

First, cubic (~10 × 10 × 10 μm^3^) azoGel micropost actuators were prepared following the fabrication procedure illustrated in Fig. 1a (see “Methods” for details). Briefly, broadly size-dispersed (from 10 nm to 250 nm, see Fig. S1) nanoparticles made from the hydrophobic photo-deformable azopolymer poly(Disperse Red 1 methacrylate – co – methyl methacrylate) p(DR1m-co-mma) were prepared using a solvent evaporation method and sterically stabilized with a low molecular weight (~13–23 kDa) polyvinyl alcohol (PVA) stabilizer^41^. To promote the formation of robust, physically cross-linked hydrogel composite networks^42^, high molecular weight (~360 kDa) polyvinylpyrrolidone (PVP) was added to the aqueous particle solutions before they were cast into a soft micro-structured template, dried, and eventually submitted to an annealing step followed by re-swelling in water. Note that a high-temperature annealing step (135 °C), in the presence of sufficient azopolymer particles, was needed to prevent dissolution of the nanocomposite structures during water re-immersion (Fig. S2).

When the swollen azoGel cubes were then illuminated from the top by a linearly polarized green (wavelength 532 nm) laser beam for 90 s, a continuously growing elongation was observed parallel to the direction of polarization (Fig. 1b and Supplementary Movie S1). Thus, the azoGel cubes inherit the polarization-guided deformation behavior of single azopolymer nanoparticles^4,43^, but on a supra-particle scale. This corroborates our recent findings at much lower strain values in a thermoplastic elastomer, where we found that the elastic matrix efficiently propagates the individual deformation of embedded azopolymer particles to the overall composite, which directionally deforms as a whole^17^. In addition to brightfield microscopy images (top row), the deformation series was also visualized in 3D using confocal microscopy images (bottom row). These show a profile with decreasing deformation along the *z*-axis (incidence direction), due to substrate attachment and the progressive absorption of the laser beam across the cube’s height^44,45^. Indeed, out-of-plane deformation gradients can be reduced by decreasing the azo-particle content and thus the absorptivity of the composite (Fig. S3). Note that when circularly polarized illumination is used instead of linear polarization, the embedded azopolymer particles are expected to undergo isotropic flattening in the plane^46^. Consequently, we observe that mushroom shapes with hats expanded along both in-plane directions are produced from the azoGel cubes in this case (Fig. S4), reminiscent of similar structures reported in pure azopolymer micropillars^47^. We also note more generally that the top surfaces of the composite actuators undergo fairly homogeneous stretching on these confocal image sequences, which indicates that the azopolymer nanoparticles are well-dispersed within the hydrogel, forming an evenly responding nanocomposite on the actuator microscale.

The unidirectional deformation behavior under linearly polarized illumination of the cubes shown above, whose dry mass consists of 50% PVA-coated azopolymer nanoparticles and 50% long chain PVP (Azo NP:(50%)PVP Gel), is quantified as a function of time in Fig. 1c. The behavior is plotted for different intensities and in both dry (dashed lines) and swollen (solid lines) states. We note that already the initial width of swollen cubes appears slightly larger due to swelling itself, which is more precisely assessed in Fig. S5. Interestingly, no noticeable cube deformations are produced in the dry state, which we attribute to the much higher, gigapascal-range stiffness of the PVP matrix in this case^48^. As the azopolymer nanoparticles are also photo-softening during illumination, it appears reasonable that their photo-deformation is inhibited by the mechanical constraint of the much larger and rigid dry matrix. In the wet, swollen state – where the stiffness of PVP is typically orders of magnitude lower – deformations are ample instead. However, while the initial deformation rates are highly dependent on the intensity shone, all curves saturate towards a maximal relative deformation of about 200%. This surprising behavior indicates a clear influence of the hydrogel matrix – and especially the swollen PVP network – on the overall photo-deformation, with restoring forces limiting the maximally reachable deformation extent to an intensity-independent saturation value.

In contrast, as a special case, we also fabricated samples without the addition of high molecular weight PVP (Azo NP Gel). In this case, the hydrophilic matrix consists only of the initially added low-molecular-weight PVA stabilizer, and the samples swell to a lesser degree (Fig. S5). Still, much larger photo-deformation is observed in the water-swollen state, and as opposed to the behavior described above, a unique deformation limit is absent for these samples. Instead, the cubes deform into extended wire-like shapes (Fig. 1d). In fact, although the structures can become wavy and difficult to track, supposedly plastic elongations of up to 1600% can be observed on Azo NP Gel cubes illuminated at higher intensities (Fig. S6 and Supplementary Movie S2). We speculate that for Azo NP Gel, in the absence of long PVP chains, only a weak physical network is created by the PVA stabilizer coating the particles, thus allowing for plastic deformation at low stresses. Experimentally, this is consistent with a strong mechanical indentation hysteresis for swollen Azo NP Gels under rheological testing (Fig. S7), as opposed to a more elastic behavior of PVP-linked gels such as Azo NP:(50%)PVP Gel. Nevertheless, additional effects are needed to explain the exotic elongations of Azo NP Gel cubes. In fact, these do not only surpass the deformations obtained with intricate approaches based on molecularly aligned materials in literature (380%)^49^, but also exceed our own pure Azopolymer Control cubes (285%) introduced below, and range even far beyond the highest elongations reported for pure azopolymer nanoparticles on solid substrates (~500%)^43^. Non-sticky behavior may be one part of the explanation. In pure azopolymers, adhesion between the photo-softened deforming azopolymer and the substrate is often evoked as a deformation-limiting factor and can e.g., pin the deforming wings of an actuator to the underlying surface^21^. Here, the hydrophilic matrix prevents direct contact between the azopolymer particles and the substrate, and despite the pronounced wing shapes seemingly touching the bottom surface (Fig. S3a), deformation continues unhampered (Supplementary Movie S2). In addition, onion-like layer-by-layer deformation across the cubes’ thickness may be imagined. Due to the high azopolymer particle content and thus the very thin, strongly deforming top layer of this sample (Fig. S3a), parts of the initially illuminated top volume may be displaced sufficiently to gradually expose deeper cube layers to the illumination. These layers can then additionally contribute to the displacement of the deforming structure’s edges, leading to the extreme apparent elongations of 1600%.

Finally, as a last control experiment, we also considered cubes made of pure hydrophobic azopolymer (Azopolymer Control). As the only sample, these cubes deformed similarly in dry and wet conditions, since they would not swell. Additionally, we observed that the wetted azopolymer residual layer decomposed in a bubble-like manner at high illumination intensity, as reported by others^50^, contrasting with the gel materials’ integrity. Also, similar to Azo NP Gel (sample without PVP), the deformation extent of Azopolymer Control cubes after prolonged illumination was dependent on intensity, the absence of a saturation limit suggesting viscoplastic behavior again. Indeed, plastic deformation behavior is well known for nanoindentation of pure azopolymer under irradiation, in the absence of restoring forces^25^. The directional deformation videos and continuous deformation graphs for Azopolymer Control cubes and all other tested samples, which further include 85% PVP dry mass-containing (Azo NP:(85%)PVP Gel) cubes, are shown in Supplementary Movie S3 and Fig. S8.

### Overwriting of previous deformations

Next, we focus our attention on the possibility of deforming the hydrogel actuators sequentially, along different directions. Such deformation overwritability appears crucial in applications wherein soft hydrogel actuators are supposed to grab or fix objects, for example, brain spheroids^51^, possibly according to multiple subsequent manipulation and release steps. Interestingly, the possibility to overwrite deformations has been previously reported for single azopolymer particles in an elastomer matrix^52^. In their pioneering work, Ryabchun & Bobrovsky invoked elastic restoring forces stored in the matrix – together with photo-softening of the embedded azopolymer particles under illumination – to explain why particles would fully recede along the initial deformation direction upon inscription of a second, differently oriented photo-deformation of the particle. Later, our group found a similar effect for macroscopic small strain deformations (<1.5%) in composites from dense azopolymer particles and a thermoplastic elastomer matrix^17^. It is worth pointing out that such behavior is not self-evident for azopolymers. On the contrary, azopolymer deformations – in the absence of restoring forces – have been shown to be superposing processes in multiple contexts. For example, applying sequential interference patterns with different orientations on thin films can create superposed square gratings^18^. Analogously, applying two sequential illumination steps with orthogonal polarization (and the same dose) to pure azopolymer pillars leads to flattened shapes, with similar extension along both deformation axes^15,17^. Overwriting, on the other hand, requires previous deformations to be actively erased whilst new ones are inscribed.

**Fig. 2:**
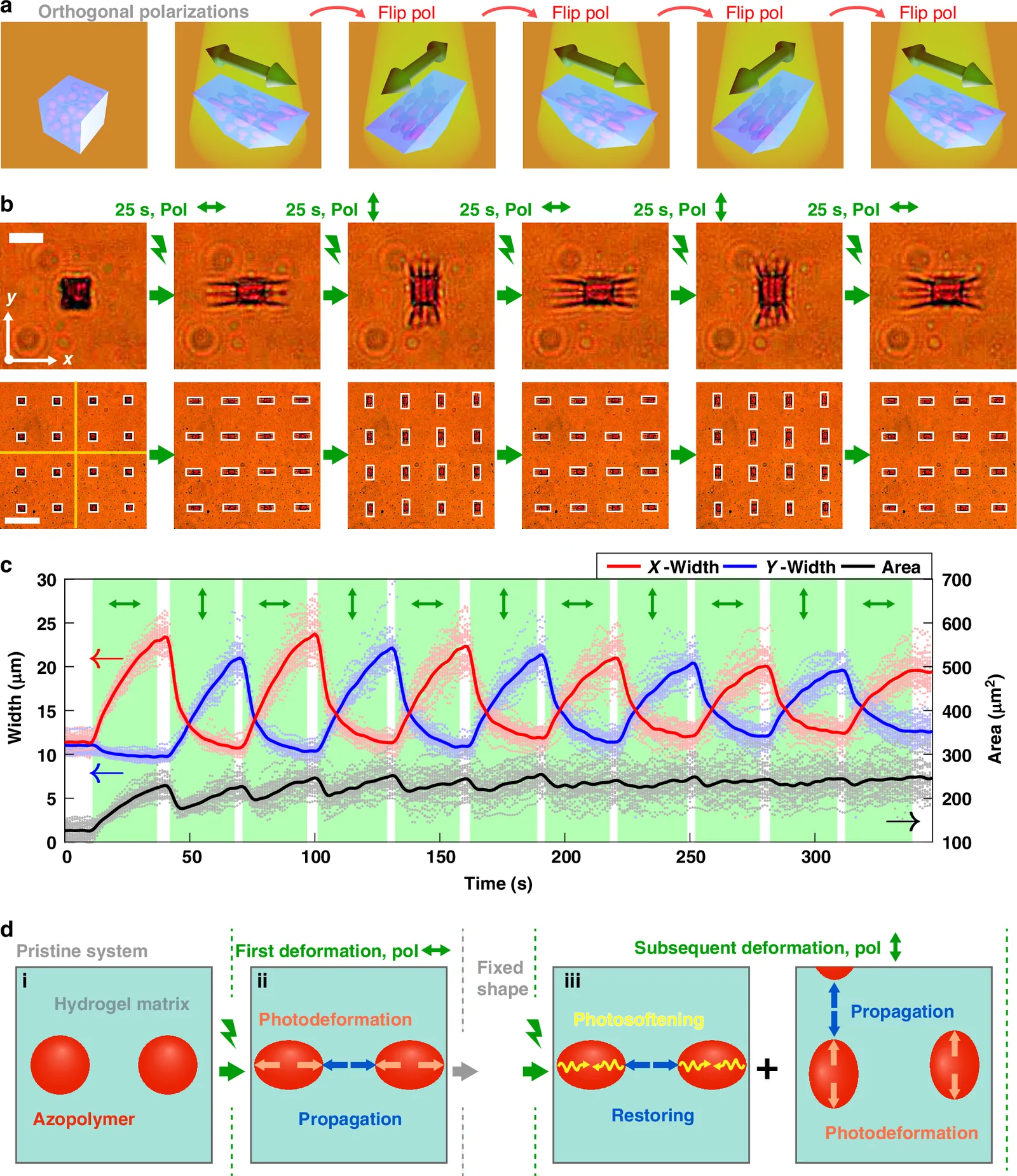
Sequential actuation of azoGel cubes using alternating polarizations. **a** Scheme of experimental procedure consisting of equally timed top-illumination steps with linear polarizations orthogonally oriented on the sample *xy*-plane. Residual layer underneath the cube omitted for visual clarity. **b** Snapshots of a single azoGel actuator cube (top row, scale bar: 10 μm) and an overview of 16 actuators (bottom row, scale bar: 50 μm) before/after the first five illumination steps. White squares (bottom row) show automatically computed cube outlines (see “Methods” section), crossing yellow lines (first bottom row image) separate four regions with individually adjusted contrast to correct for camera TAP readout, and the full experimental sequence is shown in Supplementary Movie S4. Illumination intervals: 25 s, Intensity: 39 Wcm^−2^, Material: Azo NP:(50%)PVP Gel. **c** Graph showing the continuous evolution of the average actuator *x-*width, *y-*width and area (solid lines), based on automatically tracked outlines from 32 cubes (dots), as shown in (**b**). Green background and arrows mark illumination intervals and polarization directions, respectively. **d** Schematic representation of the interactions between the azopolymer particles and the hydrogel matrix in the PVP-containing composites. Two processes are at work during overwriting, elastic recovery, and new directional deformation, which tend to be slightly temporally separated in the experiments. Note that the particles also undergo photo-softening during the first illumination (not drawn), which, however, produces no related deformation, as the surrounding pristine hydrogel matrix is unstrained

Here, to assess the overwritability of the ample azoGel deformations, we design an experiment where microcubes are illuminated by a series of equally spaced exposures, 25 s each, with alternating orthogonal illumination polarizations, as schematically depicted in Fig. 2a. The result of such a sequence being applied to Azo NP:(50%)PVP Gel cubes is shown in Supplementary Movie S4 with snapshots displayed in Fig. 2b. Figure 2c shows the tracked widths in the *x*- and *y*-direction as well as the actuator area in the sample *xy*-plane, for 32 cubes. As apparent from the graph and images, although the stretching amplitude decreases slightly along the illumination sequence, a controlled and repeatable periodic modulation of the actuator lateral size can be obtained. Interestingly, as is also apparent from the movie, the elastic recovery from previous illuminations seems to occur faster than the build-up of the next orthogonal strain, a fact that is reflected by slight area dips at each illumination onset. This observation is illustrated with additional snapshots in Fig. S9, showing how the actuator recovers most of its previous deformation before extending into a new direction during overwriting. Lastly, we found that neither this specific observation nor the overwritability in general was strongly affected by the employed light intensity. Therefore, one may adjust this parameter depending on the application, and similar overwriting amplitudes can be obtained, e.g., with shorter, high-intensity illuminations of only 5 s duration (Supplementary Movie S5, Fig. S10).

On the other hand, as expected from the differences in unidirectional deformation behavior addressed before, the elastic overwritability behavior depends strongly on the sample type. Supplementary Movie S6 shows sequences of orthogonally polarized, 10 s illuminations for all four previously introduced samples, with associated graphs plotted in Fig. S11. The worst performance pertains to the Azopolymer Control sample (pure azopolymer). In this case, the deformation amplitude quickly vanishes, the actuators’ edges seemingly deteriorate after multiple exposures, and the relative area increase is large with respect to the deformation amplitude for each illumination step. The last observation confirms the well-known plastic deformation behavior of pure azopolymer, in which subsequent deformations add up rather than overwrite each other^16,53^, thus causing persistent flattening of the material^17^. The Azo NP Gel sample also shows considerable relative area increase at each illumination step, which may again be attributed to its strong viscoplastic characteristics (Fig. S7), facilitating plastic photo-deformation. For this sample, however, the deformation amplitude does not decrease, and whilst the residual layer is prone to detachment and buckling, the actuators themselves stay intact during the experiment. Finally, the regular, PVP-containing azoGel samples both show good stability and overwritability for the 15 illumination steps tested, which we associate with their mainly elastic properties under rheological testing and thus with a strong elastic restoring force exerted on the photo-softened, irradiated azopolymer nanoparticles during overwriting^52^.

To further confirm this restoring effect by direct observation, we prepared another sample, wherein larger azopolymer particles (~1–2 μm, see Methods) were embedded in a bulk gel made from the PVP-containing Azo NP:(50%)PVP formulation. During orthogonally polarized illuminations, the photo-deformation of these larger particles can be directly observed under the microscope (Supplementary Movie S7, Fig. S12). Hence, they give an indication about the behavior of azopolymer particles in such a composite, which, for the nanoparticles used in the other experiments of this work, cannot be obtained directly. We find that the photo-deformation of these single microparticles closely mimics the behavior of the composite itself. Not only are the shapes efficiently overwritten, but also here we see a transient return to a round, less expanded shape during overwriting, before the next deformation appears. In sum of these observations, we find that two processes govern the writing and overwriting in these materials, with two-fold roles for both the embedded azopolymer particles and the hydrogel matrix (see schematic recapitulation in Fig. 2d). Namely, when pristine composites are illuminated with linearly polarized light, the photo-softened azopolymer particles deform directionally, and the hydrogel matrix propagates their deformation. As the illumination ceases, the deformed azopolymer particles recover their gigapascal rigid state^25^, and the composite deformation is fixed. Upon illumination with orthogonal polarization, the azopolymer particles photo-soften again, but this time undergo a passive remodeling by the elastic forces stored in the hydrogel matrix, in addition to the directional deformation process. We note that this restoring component is somewhat analogous to concepts encountered in shape-memory polymer blends. In some of these materials, embedded particles can be cooled to below *T*_*g*_ in a stretched composite state and then fix this overall shape – until heating above *T*_*g*_ softens the particles and allows elastic recovery mediated by the surrounding matrix^54^.

### Photo-controlled wells

**Fig. 3:**
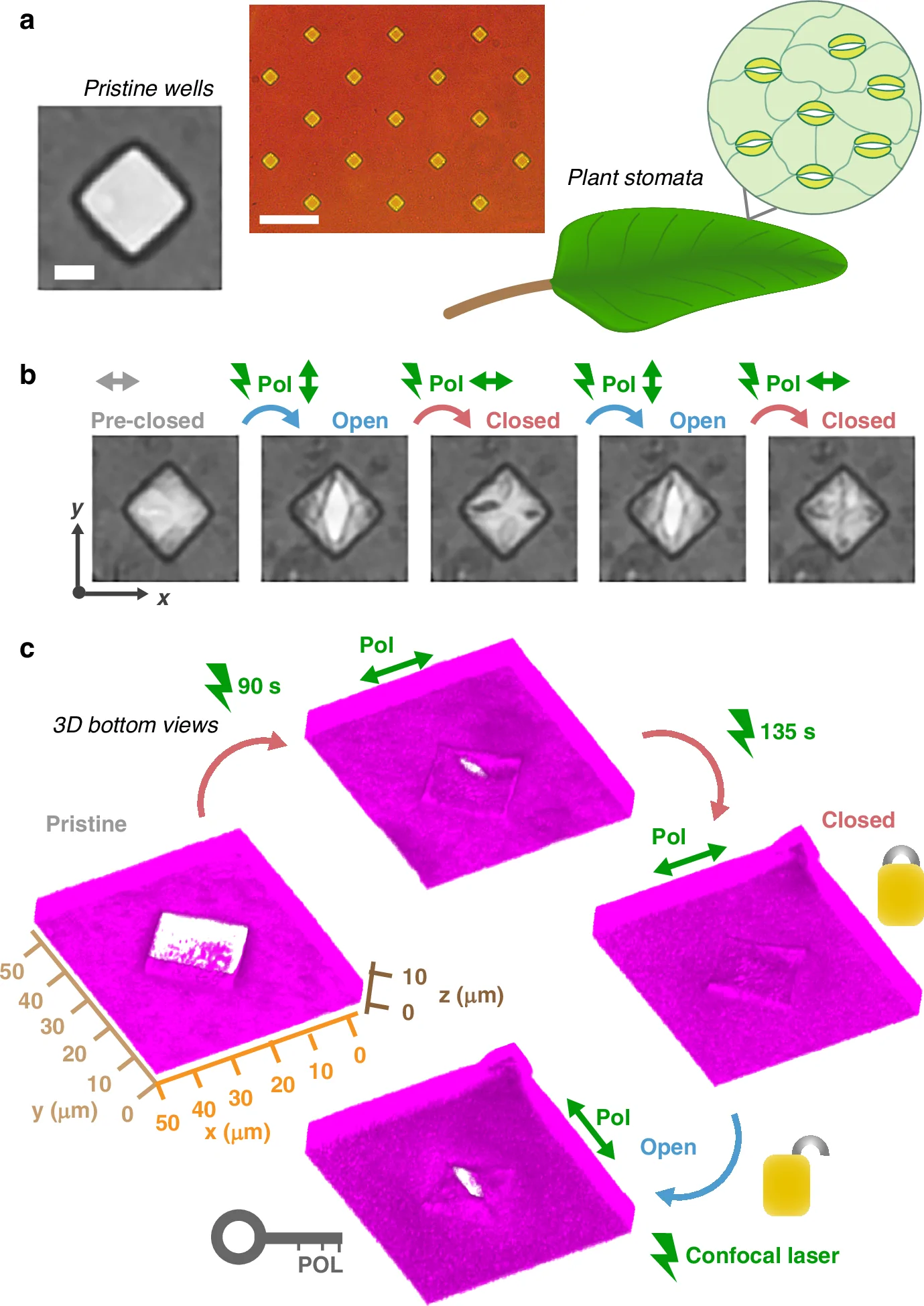
Closing and opening of stoma-like microwells. **a** Images of pristine 10 μm wide azoGel microwells as a close-up (left, scale bar: 5 μm) and as an overview image (middle, scale bar: 50 μm). On the right, a drawing of the similarly sized stomata regulating gaseous exchange in plants is shown. **b** Sequence of a 10 μm wide well that was fully closed by *x-*polarized top illumination (first image, left) being sequentially opened and re-closed using alternating orthogonal polarizations. Intensity *I* = 36 Wcm^−2^. Material: Azo NP:(50%)PVP Gel. Full sequence shown in Supplementary Movie S8. **c** 3D confocal reconstructions of pristine, partially closed, and fully closed 20 μm wells that were previously irradiated under an optical microscope (*I* = 36 Wcm^−2^). In the last step, the well is reopened directly under a confocal microscope (Supplementary Movie S9), using the confocal laser with polarization orthogonal to the closing procedure. Schemes highlight how choosing the correct polarization acts as a key to reopening the wells

As a last experiment, envisaging further application perspectives for the azoGel composites, we introduce microwells fabricated from inverse cube templates. Arrays of 10 μm wide openings in a PVP-linked gel film, reminiscent of the natural stomata regulating gaseous exchange in plant leaves^55^, are shown in Fig. 3a. Using orthogonally polarized illumination sequences, these wells can be repeatedly closed and reopened, as shown in Fig. 3b and Supplementary Movie S8. Worth to highlight that such behavior – and especially the reopening of wells – is only possible due to the combination of two properties of the composite material, namely a good deformation overwritability and a non-sticky behavior contrasting with the self-healing properties of pure azopolymer. Self-healing properties in pure azopolymers stem from the fact that adjacent polymer volumes can merge under light-induced reflow, fusing into a single piece of material. This effect has, for example, been used to demonstrate the healing of azopolymer cracks using appropriate illumination, even restoring conductivity across the healed crack when the azopolymer was initially doped with conductive fillers^20^. In the framework of the wells here, such an effect would be detrimental instead, since the closing well surfaces would risk fusing together and becoming inseparable by subsequent illumination. Instead, the non-sticky hydrogel matrix surrounding the azopolymer particles prevents their direct contact, even if different actuator parts are touching, and thus permits subsequent reopening of fully closed wells.

In addition, the different stages of well closing and opening can be visualized in 3D using confocal microscopy (Fig. 3c). Besides confirming well closure, these images, shown from a bottom perspective, visualize the enclosed space underneath the deformed top layer. In particular, the initial square-well shape is still visible on the substrate side, while the top of the well is closed. This is due to the previously discussed effect of absorption along the *z-*axis, which ensures that only the top part (facing the incident light) deforms substantially. However, as the depth of deformation of the AzoGel composites can be readily tuned by the azopolymer concentration (Fig. S3), the thickness of the closing layer may also be tuned according to the application prospect. Furthermore, only the correct polarization (orthogonal to the one used for closing) can unlock and open the well. The illumination polarization thus acts as a key, and targeting the opening of specific wells can be imagined even with (polarized) blanket exposure of entire samples. Finally, we point out that given the typical moderate intensities needed to trigger photo-deformation in the composites, the opening of the well in Fig. 3c was performed by directly using the built-in 10 mW, 555 nm laser of the confocal microscope. Hence, in this case, actuation and 3D well surface imaging (Supplementary Movie S9) were combined directly on a commercially available instrument.

## Discussion

In summary, we introduced azopolymer nanoparticles as deformation drivers within hydrogel matrices. We successfully demonstrated that the large, directional deformations of individual azopolymer nanoparticles, described in previous studies^43^, can be transferred to micrometric composite gel actuators. Compared to pure azopolymer, the azoGels were found more suitable for prolonged illumination in aqueous environments and consistently deformed to greater extents at the lower illumination intensities used (11 Wcm^−2^). Both these aspects are favorable for the development of biotechnological devices. Although only simple, physically cross-linked gel matrix formulations were introduced here, the results also suggest that properties such as the deformation depth, overwritability, and amplitude may easily be tuned and, in some cases, traded against each other via composition changes. In Table 1, some of the key photo-mechanical properties elaborated in this article are summarized for the four different sample types studied. In brief, we observed during mechanical nanoindentation studies that the addition of high molecular weight PVP rendered the samples’ response considerably more elastic than that of samples lacking PVP, which we attributed to stronger physical cross-linking. Correspondingly, the photo-deformation of PVP-containing samples was limited to a maximal value – presumably due to restoring forces prohibiting further deformation – while PVP-free samples underwent extreme deformations which we have not encountered in the context of azopolymers before. In turn, PVP-free samples showed plastic deformation behavior during overwriting attempts with deformations accumulating, whilst PVP-containing samples showed high overwritability owing to an elastic restoring effect.

**Table 1:** Summary of (photo-)mechanical properties

Sample	Azopolymer Control	Azo NP Gel	Azo NP:(50%)PVP Gel	Azo NP:(85%)PVP Gel
Properties				
Swelling ratio (ΔV/V) (%)	n.a.	74 ± 34	186 ± 53	157 ± 49
Young modulus (MPa)	~3400^a^	4.6 ± 0.2	1.15 ± 0.05	0.18 ± 0.01
Nanoindentation hysteresis (@100 nm)	n.a.	Large	Detectable	None
Stability in water under illumination	No	Partially	Yes	Yes
Directional deformation extent (low intensity) (*I* = 11 Wcm^−2^, *t* = 90 s) (%)	53 ± 1	80 ± 3	142 ± 8	128 ± 6
Directional deformation extent (high intensity) (*I* = 126 Wcm^−2^, *t* = 90 s) (%)	285 ± 23	1270 ± 347	207 ± 3	197 ± 8
Cube (*h* ≈ 10 μm) deformation depth (*I* = 35 Wcm^−2^, *t* = 90 s) (μm)	n.a.	4.2 ± 0.4	6.8 ± 0.4	>8.8
Overwritability (15 orthogonal deformations, *I* = 39 Wcm^−2^, *t* = 10 s)	Poor	Partial	Good	Good

Swelling ratio: volume change estimated from 3D confocal images in wet and dry conditions (Fig. S5). Young modulus: expressed as the mean ± sem: rheological nanoindentation measurements in 6 different sampling points for each azoGel composition (Fig. S7). Note that the values represent averages, not adjusted for the varying density of rigid azopolymer particles Nanoindentation hysteresis: based on qualitatively assessing individual rheological curves (Fig. S7). Film stability: occurrence of substrate detachment, degradation, or bubbles under wet illumination. Maximal directional deformation: saturation values from deformation curves in Fig. 1 and Fig. S8. Deformation depth: full width at half maximum of deformed cube depth profiles (Fig. S3). Overwritability: qualitative assessment based on structural integrity, maintained amplitude, and relative area expansion (flattening) during orthogonal photo-deformation sequences (Fig. S11). Samples: Pure azopolymer (Azopolymer Control), PVA-coated azopolymer nanoparticle gels (Azo NP Gel), and gels with added PVP (Azo NP:(50/85%)PVP Gel). Parameters: *I =* intensity, *t =* time. *h =* initial height

^a^Non-illuminated azopolymer is similar to poly Disperse Red 1 methacrylate p(DR1m), value from literature^25^.

In this regard, an important fundamental difference with respect to previously reported hydrogels undergoing anisotropic photo-deformation is the following: To date, these actuators were usually built upon the incorporation of aligned nanomaterials into stimuli-responsive hydrogels^36,37,39^. While the fabricated alignment of the nanomaterials would determine the deformation direction in the final device, the actuation itself would thus rely on the photo-response of a suitable hydrogel matrix, often poly(N-isopropylacrylamide) (PNIPAM). Here, instead, the deformation direction can not only be decided and altered during use of the composite, but it is the anisotropic deformation of the nanomaterials (azopolymer nanoparticles) themselves, which drives the deformation. The matrix is therefore merely a passive element which serves to propagate the deformation, to provide elastic restoring forces, and to interface the azopolymers with the surrounding aqueous environment. Rather than being limited to a specific type of matrix material, we therefore foresee possibilities to explore a wide range of different and possibly application-specific hydrogels. For example, covalently cross-linked, e.g., acrylic hydrogels may be promising matrix candidates. In this case, one would be able to tune the elastic properties by tuning the cross-linker ratio of the gel, without indirectly altering, e.g., the azopolymer particle content. This would be a relevant modification both for fundamental and applied studies. In addition, block copolymers using azobenzene-containing blocks have been developed in the past^56–58^, and one could imagine designing amphiphilic ones that may directly phase-separate into similar photo-deformable azopolymer-hydrogel composites. More generally, doping azopolymer particles into different types of hydrogels, material-specific fabrication approaches, and biofunctionalization strategies may be exploited^59^, and the external matrix chosen according to the application requirements.

Application-wise, employing photo-deformable hydrogel pillars and wells as shown in this work may be of interest in various challenges of biotechnology and microfluidics. For example, arrays of photo-deforming azopolymer pillars have previously been used as on-demand cell-instructive substrates in biology^12,13^, and equipping such structures with overwritability should allow for full spatiotemporal control. In microfluidics, arrays of pillars can be used to steer flows as well as to control the deterministic lateral displacement of particles to be separated from a stream, with anisotropic pillar shapes and orientations having been proposed to precisely tailor devices^60,61^. Also here, dynamic pillar elements may open further possibilities. Regarding hydrogel wells in biotechnology, we note that a growing body of work uses such wells to culture single cells^62^, cell aggregates^63^, or organoids^64^ in precisely defined microenvironments. Added functionalities in these works include the extraction of extracellular vesicles through the manual breaking of an oil seal covering the wells, the manufacturing of non-trivial and closed 3D well shapes to determine organoid development, and the release of cell aggregates via a stimulus-induced swelling response of wells. By means of their localized and thickness-tunable directional deformation, wells, as shown in this work, may perform such functions dynamically and without contact. Finally, in microfluidics, there are several examples of photo-responsive hydrogel valves^65,66^, sometimes based on micropores that resemble our wells^67^, which usually rely on optically induced swelling/deswelling responses. In this context, the possibility of fully closing wells and retaining any produced shape automatically in the dark may become interesting. That said, we foresee that the exploration of various hydrogel matrix formulations will also permit the fabrication of more complex geometries than wells and pillars, such as 3D cell scaffolds^68^ or intricate 3D-printed hydrogel actuators^69^.

With this prospect of obtaining even more complex pristine azoGel actuator structures in 3D, it is worth briefly discussing the obtainable degrees of freedom in their deformation. Traditionally, works on azopolymer surface structuring have focused heavily on aspects such as optimizing the azopolymer molecular weight and film thicknesses^70,71^, on the polymer chemistry^72,73^, on refined illumination setups^8,74^, and sometimes on surface reconfigurability^75,76^. This has permitted strong advances in the obtained surface relief heights, the diffraction efficiencies, and in accurate surface profile shaping. Still, the local degree of freedom is essentially equal to one and corresponds to the local height of the surface profile, even if the directional deformations associated with polarization patterns were involved in the formation of said profile^77^. By starting from overwritable pillars, the degrees of freedom theoretically increase to three, corresponding to the extent of directional pillar deformation, its angular orientation, as well as simultaneous deformation of the minor axis, which can in principle be obtained through the use of elliptical polarization^46,47^. More so, if the illumination is not limited to normal incidence, but can hit a protruding structure from any incidence angle, the number of available deformation degrees of freedom increases to five^78^. To this, one may add the possibility of providing illumination only at specific points of the entire actuator. On the other hand, such freedom of incidence direction and target locations would also require advanced illumination set-ups.

In the context of illumination strategies and how to practically deploy the potential of the azoGel actuators introduced here, we point out that the illumination is not limited to Gaussian laser beams. In fact, no coherent illumination is needed to deform azopolymers^44^, and compact devices with tailored incidence directions and spatial control may even rely on micro-LED arrays or integrated waveguides to address and steer the actuators^79^. Conversely, polarized blanket LED exposure, e.g., to selectively address pre-closed wells, or the use of the built-in components of conventional confocal laser microscopes^13^ may constitute cheaper and very convenient ways to control the photo-deformations in biological application scenarios. Finally, we note that in-situ patterning approaches for large 3D cell scaffolds usually rely on multiphoton illumination for localized functionalization^80^. In this regard, it is worth noting that the directional deformation of the azopolymer type used here has also been demonstrated using two-photon inscription^81^.

## Methods

### Particle fabrication

An azopolymer solution of 10 mg/mL poly(Disperse Red 1 methacrylate-co-methyl methacrylate) p(DR1m-co-mma) (purchased from Sigma Aldrich, ~15 mol% dye monomer) in chloroform is prepared. Subsequently, the azopolymer solution is combined with an aqueous solution containing 0.5 wt% polyvinyl alcohol (PVA, purchased from Sigma Aldrich, MW: 13–23 kD, 87–89%, hydrolyzed) at a volume ratio of 1:12.5. The immiscible two-phase system is emulsified in a closed container immersed in an ultrasonic bath (Elmasonic P30H, 37 kHz, 100%) for several hours, with temperature constantly kept between 45° and 55 °C, and under occasional manual shaking, until appearing fully homogeneous by eye. Particles are obtained from the emulsion by transferring the system to an open beaker and slowly raising the temperature of the water bath until the chloroform is evaporated (final temperature 80 °C). Excess PVA is removed from the particle suspension by centrifuging and re-suspending the particles in pure DI water three times. The supernatant of all centrifugation steps is kept apart and measured against a 0.0025 mg/mL reference concentration in acetone using a UV-VIS spectrometer, to estimate the total loss of azopolymer during purification (~18% loss after three rounds, see Fig. S13). Then, the water amount during the last resuspension is adjusted to obtain a small volume of concentrated ~30 wt% azopolymer particle solution, which can be used as a precursor for the AzoGel fabrication below. In the case of the larger azopolymer particles used for the individual particle deformation studies in Fig. S12, a slightly modified protocol with a polyvinylpyrrolidone (PVP) precursor solution and different proportions was utilized as reported previously^46^.

### AzoGel fabrication

The particle precursor solution obtained above, consisting of 30 wt% azopolymer particles coated with PVA, is either used as such (for Azo NP Gel samples) or mixed with an aqueous solution containing 10 wt% polyvinylpyrrolidone (PVP, purchased from Sigma Aldrich, MW: 360 kD) at the proportion required to obtain the desired ratio of solid content between nanoparticles and PVP (for Azo NP:(50%)PVP and Azo NP:(85%)PVP samples). The precursor solutions are degassed in a vacuum desiccator and then dispersed between a clean (acetone and O_2_ plasma treated) microscopy glass slide and polydimethylsiloxane (PDMS, Sylgard 184 purchased from Dow Corning) master molds. The master molds contain the inverse microstructures to be printed, i.e., negative replicas of 10 × 10 × 10 μm^3^ cubes/well arrays previously fabricated by conventional lithography with SU-8 photoresist. After drying, the PDMS mold is peeled off, and the microstructures are annealed on a hotplate in the dry state for 10 min at 135 °C, which exceeds the glass transition temperature of p(DR1m-co-mma) (Tg~105 °C, according to the provider). Before utilization as composite gels, the structures are routinely immersed in DI water for >1 h. Finally, control azopolymer samples are fabricated directly from a 30 wt% solution of p(DR1m-co-mma) in anisole, using the same PDMS master mold imprinting procedure as for the composite gels, without annealing.

### Particle characterization

To obtain an estimate of the particle size distribution, diluted droplets of aqueous particle solution are dried on a piece of silicon wafer. The substrate is then sputter-coated with 10 nm of platinum-palladium. Images are obtained using a scanning electron microscope (SEM Apreo, Thermo Fisher) using the T1 detector (back-scattered electrons), 5 kV acceleration voltage, and a 50 pA electron current (magnification 30000×, image size 4096 × 3536 pixels, 1.1 nm/pixel). Particle outlines and sizes are detected using ImageJ by the following procedure: *Convert to 8* *bit ◄ Adjust Contrast (160–255) ◄ Auto Local Threshold v1.11 (“mean”, 15 pixels, P1* = *0) ◄ Convert to Mask ◄ Filter (“median”, 5 pixels) ◄ Fill Holes ◄ Watershed ◄ Analyze Particles (Circularity: 0.6–1.0)*. Finally, an average diameter is computed based on the detected area for each particle, and 20 nm are subtracted to account for the metal coating. The procedure is applied to 8 images from distinct regions of the sample (example images shown in Fig. S1), leading to the detection of 3491 particles. Finally, to obtain an estimate of the volume distribution the absolute particle counts were reweighted using the following equation (which leaves the total amount of renormalized particle counts unaltered):

$${h}_{i}=\frac{{n}_{i}\cdot {V}_{i}}{\sum _{j}{n}_{j}\cdot {V}_{j}}\cdot \begin{array}{cc}{\sum }_{j}{n}_{j}, & {V}_{i}=\frac{4}{3}\pi \cdot {r}_{i}^{3}\end{array}$$

where *n*_*i*_ designs the net particle count in the *i*th histogram interval, *h*_*i*_ designs its reweighted value used for the histograms in Fig. S1f, and *r*_*i*_ designs the radius of the particles at the center of the *i*th interval, which is used to calculate the characteristic Volume *V*_*i*_. The distributions were plotted on a logarithmic scale.

### Image acquisition procedures

For brightfield actuation videos, the sample glass slides are turned upside down and glued to the bottom of a microscopy dish using 200 μm of spacer tape, with the actuator structures facing downwards (towards the dish surface). The space between the dish and the sample glass slides is then filled with DI water using capillary forces. A 532 nm continuous wave laser diode with tunable output power is used to illuminate the samples from below (i.e., hitting the top surface of the bottom-facing micro-actuators). Optical elements placed in the laser path include lenses to adjust the spot size and polarization elements (polarizer, quarter- and half-wave plates) to control the polarization of the incident laser illumination. The videos are acquired in transmission configuration, with a broadband Tungsten-Halogen lamp providing brightfield illumination and a microscope setup consisting of an infinity-corrected 20×/0.4 NA air objective, a 550 nm long-pass filter, a tube lens, and a color CCD camera (Thorlabs) mounted on the opposite side of the laser and brightfield illumination sources. Note that although the long-pass filter removes direct laser illumination, high intensity creates fluorescence from the azopolymer, which is seen as a brightness change coinciding with the laser spot. For some single image acquisitions of final deformed structures, such as for the snapshots in Fig. 1b, the long-pass filter is removed to enhance contrast in the absence of laser illumination.

For 3D profiles of the actuators, a confocal microscope (Zeiss Airyscan 700) is used. The samples are placed in a dish with the actuator structures facing upwards and imaged using a 40×/1.0 NA water-dipping objective. In general, the 639 nm laser is used for 3D scans to avoid strong absorption of laser light by the azopolymer structures, thus relying on the scattering from the particle-gel structure for signal. The voxel size is 0.16 × 0.16 × 0.2 μm^3^. In Supplementary Movie S9, the 555 nm laser is used instead for continuous 3D scans, to both induce deformation (well opening) and perform 3D imaging of the top surface (here, the signal depth is absorption-limited). In Fig. S5a, the dry cube is imaged using a fluorescence signal at 580 nm (excitation wavelength 546 nm), due to low scattering in the dry case. When confocal image slices of cubes are segmented, as in Fig. S5 to determine swelling volumes, or in the context of Fig. S3 to determine the deformation depth, the following procedure in *ImageJ* is applied in general: First, the *z-*extent of the actuator is estimated by eye, determining an approximate first and last slice. The intensities are then normalized to the maximum found on an intermediate slice at half the height of the actuator. The precise height of the actuator is then set between an onset slice with clear signal detection and a final slice with a substrate background signal of equal intensity to the first slice. All slices are threshold-segmented at 20% of the maximal intensity previously obtained, leading to binary images of each actuator slice. Then, we apply the sequence *Maximum 3D (2px, 2px, 4px) ◄ Fill holes ◄ Median (2px) ◄ Erode* to detect smooth and continuous areas pertaining to the actuator on each slice. In the context of the Azo NP Gel cubes deformation depth (Fig. S3d, left), due to strong signal and proximity of the substrate background, the procedure is simplified to *Erode -> Median (2px) -> Dilate*.

### Automated image analysis

A deep learning-based segmentation tool, Cellpose 3.1.1.1, is used for the automated image analysis. Firstly, the brightfield actuation videos are cropped to only the region of interest, normalized for brightness, and converted to images at 1 Hz. Next, one of the images is loaded into the Cellpose graphical interface and manually segmented with respect to azoGel cube regions for retraining models (learning rate: 0.1, weight decay: 0.0001 or 0.001, number of epochs: 100 or 200). The image loading and manual segmentation are repeated for approximately 50 images until the model is sufficiently accurate to segment regions. Retrained models are prepared for each material composition and illumination pattern. All converted images are then subjected to automatic segmentation of azoGel cube regions in Cellpose using the corresponding retrained model for each movie. Segmented areas are extracted directly, while widths and heights are calculated from bounding rectangles drawn around the segmented regions. For orthogonal polarization sequences (Fig. 2 and Fig. S11), a low-pass filter with a cutoff frequency of 0.2 Hz is applied to the acquired data of areas, widths, and heights. All procedures, except for model re-training, are performed in Python.

### Rheological measurements

The Young’s modulus of the different azoGel composites was measured by a Bioscope (Bruker) atomic force microscope equipped with a Nanoscope IIIa controller. Silicon nitride pyramidal AFM tips (Bruker DNP) with a half angle of about 20° and a tip radius of curvature of about 10–20 nm were used. Different cantilevers were exploited according to the different rigidity of the sample (nominal spring constants: *k* = 0.06 N/m and *k* = 0.12 N/m). The effective spring constants of the cantilevers were measured by exploiting the thermal noise method^82^. To obtain a statistical analysis, for each sample, 6 sample points, respectively separated by at least 100 μm, were analyzed, and for each sampling point, a number of force curves ranging from 20 to 40 were acquired. Small indentation values of about 100 nm (corresponding to an applied force of less than 1 nN) were applied in order to limit the contributions coming from the underlying glass support. Vertical scan speeds of 2 μm/s were applied. The Nanoscope Analysis 1.5 software package was used to fit the Hertz model for pyramidal tips to force curves in order to obtain Young’s modulus for each curve. A Poisson ratio of 0.49 was assumed for all fits.

## Supplementary information

Supplementary Information

Movie S1

Movie S2

Movie S3

Movie S4

Movie S5

Movie S6

Movie S7

Movie S8

Movie S9

## Data Availability

The data and analysis codes that support the findings of this study can be made fully available.
