# Supplementary material for: Photo-guided azopolymer hydrogel actuators
Source: Light Sci Appl. 2026 Sep 17;15:383. doi: 10.1038/s41377-026-02411-5 (PMC13582930; doi:10.1038/s41377-026-02411-5)
Supplement: Supplementary file 1 [file 41377_2026_2411_MOESM1_ESM.pdf]

Supplementary Information

Photo-guided Azopolymer Hydrogel Actuators

David Urban[1,2,3]* Ryota Toyohara[4], Marcel Rey[5], Daniele Martella[6,7], Dag Roar Hjelme[1], Andrea Alessandrini[8,9], Toshiro Ohashi[4], Emiliano Descrovi[2]*

Affiliations:

[1]Department of Electronic Systems, Norwegian University of Science and Technology; Trondheim, NO-7491, Norway.

[2]Department of Applied Science and Technology, Politecnico di Torino; Torino, 10129, Italy.

[3]Microsystems and Nanotechnology (MiNaLab), SINTEF Digital; P.O. Box 124, Blindern, Oslo, 0314, Norway.

[4]Faculty of Engineering, Hokkaido University; Sapporo, 060-8628, Japan.

[5]Institut für Physikalische Chemie, Universität Münster; Münster, 48149, Germany.

[6]Department of Chemistry, Università di Firenze; Sesto Fiorentino, 50019, Italy.

[7]European Laboratory for Non-Linear Spectroscopy (LENS); Sesto Fiorentino, 50019, Italy

[8]Department of Physics, Informatics and Mathematics, University of Modena and Reggio Emilia; Modena, 41125, Italy.

[9]CNR-Nanoscience Institute-S3; Modena, 41125, Italy

*Corresponding author. Email: david.urban@sintef.no (D.U.); emiliano.descrovi@polito.it (E.D.)

Contents:

Descriptions of Supplementary Movies S1 to S9
Supplementary Figures S1 to S13

Supplementary Movie Descriptions

Movie S1

Linear deformation of two azoGel cubes with PVP in response to 90 s of illumination linearly polarized along the *x*-axis (horizontal). Intensity $I = 35$ Wcm^{-2}. Material: Azo NP:(50%)PVP Gel. 10x accelerated.

Movie S2

Linear deformation of two azoGel cubes without PVP in response to 90 s of high intensity illumination linearly polarized along the *x*-axis (horizontal). Intensity $I = 126$ Wcm^{-2}. Material: Azo NP Gel. 10x accelerated.

Movie S3

Linear deformation of four types of azoGel cubes (Azopolymer Control, Azo NP Gel, Azo NP:(50%)PVP Gel, Azo NP:(85%)PVP Gel) in response to 90 s of illumination linearly polarized along the *x*-axis (horizontal) with different intensities (11 Wcm^{-2}, 61 Wcm^{-2}, 126 Wcm^{-2}). Bottom row shows deformation attempts in the dry case, where only Azopolymer Control attains large deformation. Cube deformation and residual layer states are most easily appreciated after illumination is shut (final frames). The continuous quantification of deformations is shown in Figure S8. 5x accelerated.

Movie S4

Sequential orthogonal deformations of 16 azoGel cubes with PVP in response to 25 s illumination steps linearly polarized with alternating polarization direction along the *x*-axis/*y*-axis (horizontal/vertical). Illumination steps are spaced by 5 s illumination off time. Frames on the left and on the right show the same movie without and with tracked cube bounding box overlay (white) respectively. Note that contrast is adjusted individually in four equally sized quarters of the field of view to correct for camera TAP readout (raw data available). Intensity $I = 39$ Wcm^{-2}. Material: Azo NP:(50%)PVP Gel. 10x accelerated.

Movie S5

Sequential orthogonal deformations of 2 azoGel cubes with PVP (center) in response to 5 s illumination steps linearly polarized with alternating polarization direction along the *x*-axis/*y*-axis (horizontal/vertical). Illumination steps are spaced by 10 s illumination off time. Intensity is $I = 126$ Wcm^{-2}. Material: Azo NP:(50%)PVP Gel. 10x accelerated.

Movie S6

Sequential orthogonal deformations of 16 cubes for four types of samples (Azopolymer Control, Azo NP Gel, Azo NP:(50%)PVP Gel, Azo NP:(85%)PVP Gel) in response to 10 s illumination steps linearly polarized with alternating polarization direction along the *x*-axis/*y*-axis (horizontal/vertical). Illumination steps are spaced by 10 s illumination off time. Contrasts adjusted similar to Supplementary Movie S4 for each sample to correct for camera TAP readout (raw data available). Intensity $I = 39$ Wcm^{-2}. Continuous quantification of deformations is shown in Figure S11. 10x accelerated.

Movie S7

Sequential orthogonal deformations of bulk azoGel films with PVP, residing on a glass substrate and doped with large PVP-coated azopolymer microparticles. The movie consists of two parts from independent acquisitions. In the first part, the movie is first shown in overview mode, then repeated while zooming in on two prominent particles and then repeated again while tracking the two particles using ImageJ (details in Figure S12). Illumination steps are 6 s. Intensity $I = 66$ Wcm^{-2}. In the second part, the movie is shown in overview mode only, but again features (at least) two prominent particles. Here, illumination steps are 8 s. Intensity $I = 118$ Wcm^{-2}. In both cases, the illumination steps are linearly polarized with alternating polarization direction along the *x*-axis/*y*-axis (horizontal/vertical). Material: Azopolymer microparticle-doped Azo NP:(50%)PVP Gel. 5x accelerated (both parts).

Movie S8

Sequential orthogonal deformations of azoGel film hosting a 10 µm wide microwell in response to illumination steps linearly polarized with alternating polarization direction along the *x*-axis/*y*-axis (horizontal/vertical). Intensity $I = 39$ Wcm^{-2}. Material: Azo NP:(50%)PVP Gel. 5x accelerated.

Movie S9

Opening under the confocal microscope of a previously closed 20 µm microwell with polarization orthogonal to the closing procedure. Intensity (confocal laser) $I = 6$ Wcm^{-2}. Wavelength (confocal laser): 555 nm. Scanned volume: 53 x 53 x 15 µm. Material: Azo NP:(50%)PVP Gel. 330x accelerated. Looped three times for visualization purposes.

Supplementary Figures

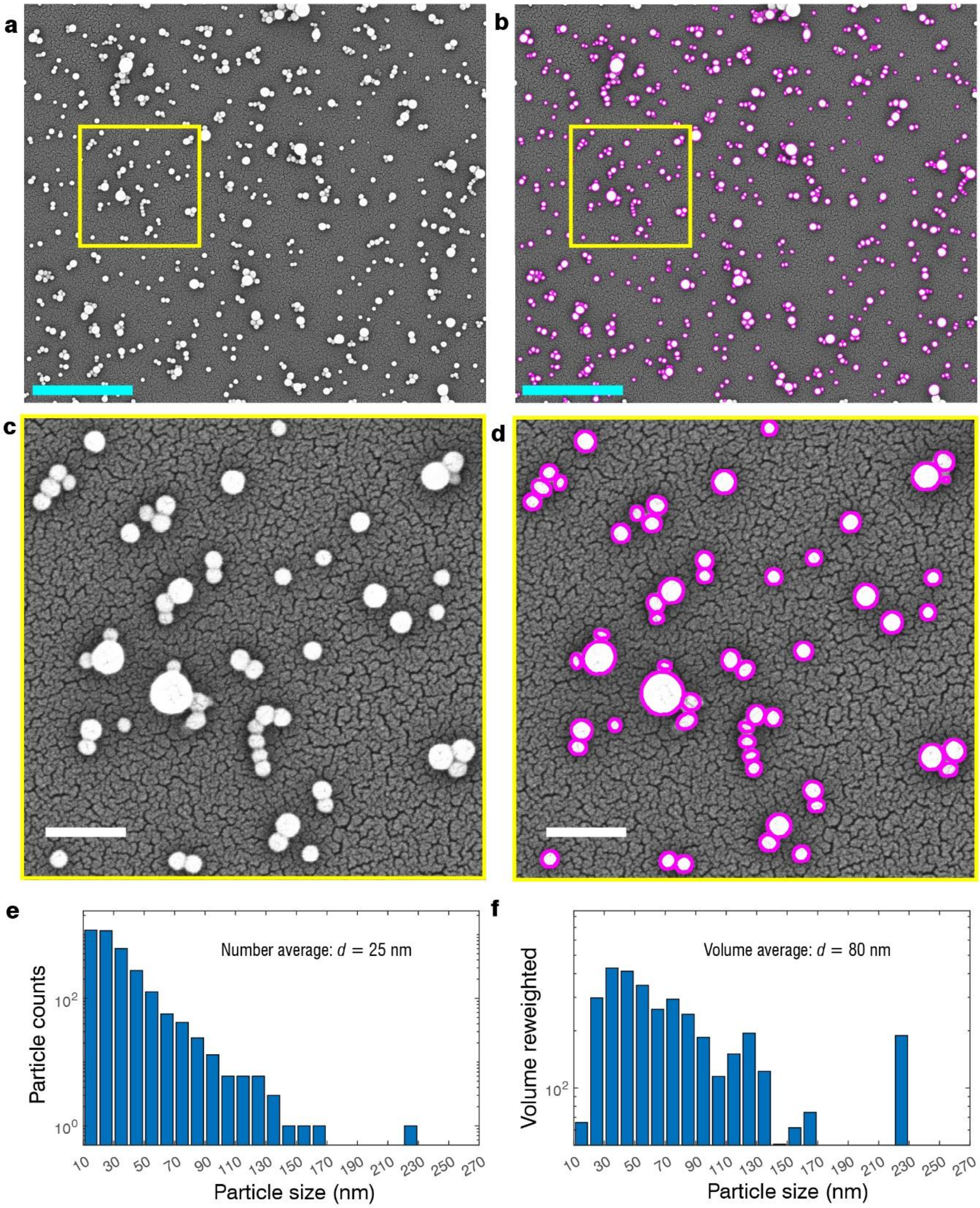

Figure S1. Azopolymer nanoparticle size distribution. a) One of eight wide SEM images used to assess particle size statistics. Yellow frame: inset magnified in c/d. Scale bar (cyan): 1 µm. b) Same as in a, with detected particle outlines overlaid in magenta. c/d) Magnified sub-region of the wide images in a/b. Scale bars (white): 200 nm. e) Particle counts within each 10 nm diameter interval. f) Particle counts re-weighted with respect to the average volume occupied by the particles in each interval, showing that larger particles, although scarce, can occupy a relevant volume fraction. These particles cause the optically scattering properties of the composite.

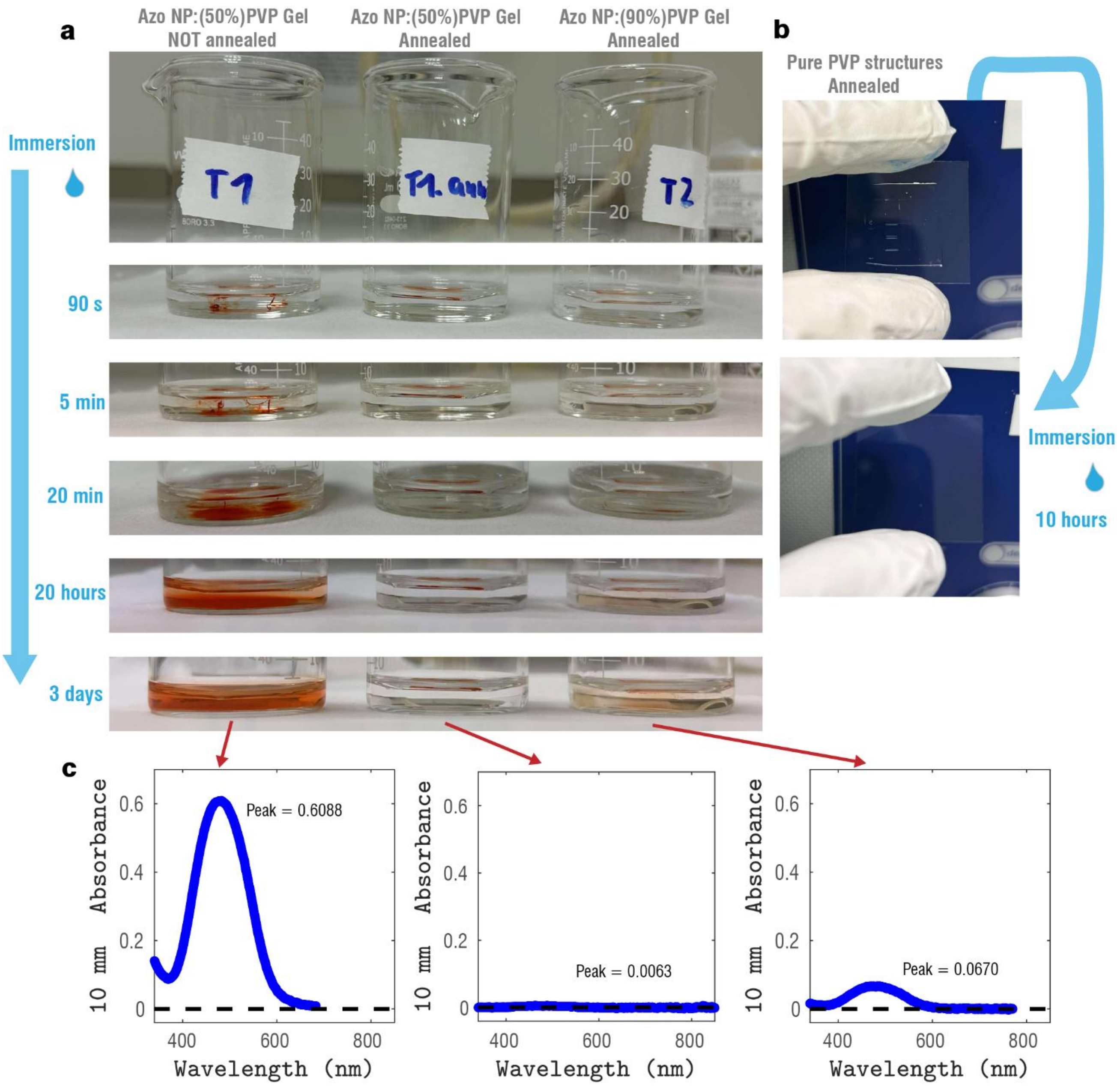

Figure S2. Dissolution test for physically cross-linked samples. a) Photographs taken at different delays after immersion in water of microstructures molded from azoGel composite onto glass substrates. These show that the microstructures of the non-annealed Azo NP:(50%)PVP sample (left) detach instantly and start dissolving, as opposed to the 3-day stable Azo NP:(50%)PVP Gel structures in the annealed case (middle). At very high PVP (and thus low azopolymer particle) contents, partial dissolution after prolonged timespans occurs despite annealing, see Azo NP:(90%)PVP sample (right). In this case we conjecture that too few particles (<10 wt%) are present to efficiently cross-link the composite upon annealing. b) Microstructures made from (water-soluble) PVP only dissolve readily, regardless of annealing. c) UV-Vis absorption spectra of the immersion liquids shown in a (3 days immersion). The spectral shape corresponds to the absorption spectrum of the azopolymer (see also Figure S13), thus indicating the presence/absence of azopolymer from dissolved microstructures. Note that whilst the right sample has only 5 times less azopolymer content than the other two samples, the peak value is 10 times less than in the fully dissolving sample on the left, indicating partial dissolution. This is consistent with visual inspection of microstructure remains on the glass slide.

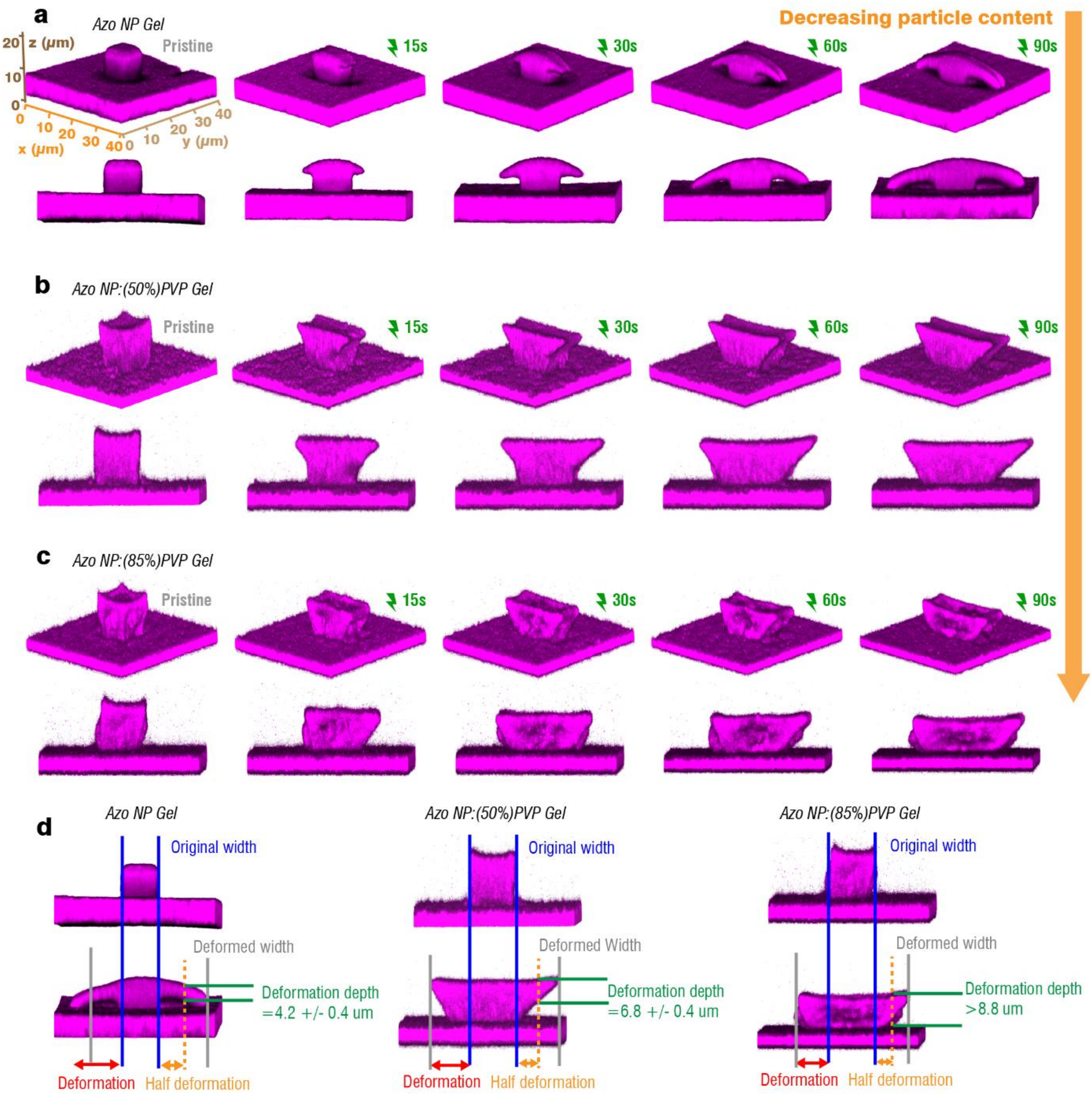

Figure S3. Deformation profiles for different composites. a) 3D confocal images (top row: top-side view, bottom row: side view) of an Azo NP Gel micro-actuator cube (consisting of PVA coated azopolymer particles only) subjected to linearly *x*-polarized illumination (wavelength 532 nm) for increasing illumination times (left to right) at an intensity of 35 Wcm^{-2}. Illumination is incident from the top (along the *z*-axis). A thin top layer is observed to be the mainly deforming part for this sample. b, c). Same as a, but for samples where the PVA-coated azopolymer particle content is diluted by 50 wt% and 85 wt% PVP respectively, as indicated. As the content of strongly absorbing azopolymer particles decreases, the deformation depth increases, leading to a less pronounced gradient of the deformation. Scales are equivalent in panels a-c. d) Schematic of the deformation depth definition as the thickness of the actuator at which half the maximal deformation (with respect to an undeformed actuator) is measured. Exact numbers are extracted from threshold-segmented confocal slice images, analogously to Figure S5, with an error margin of two slice thicknesses.

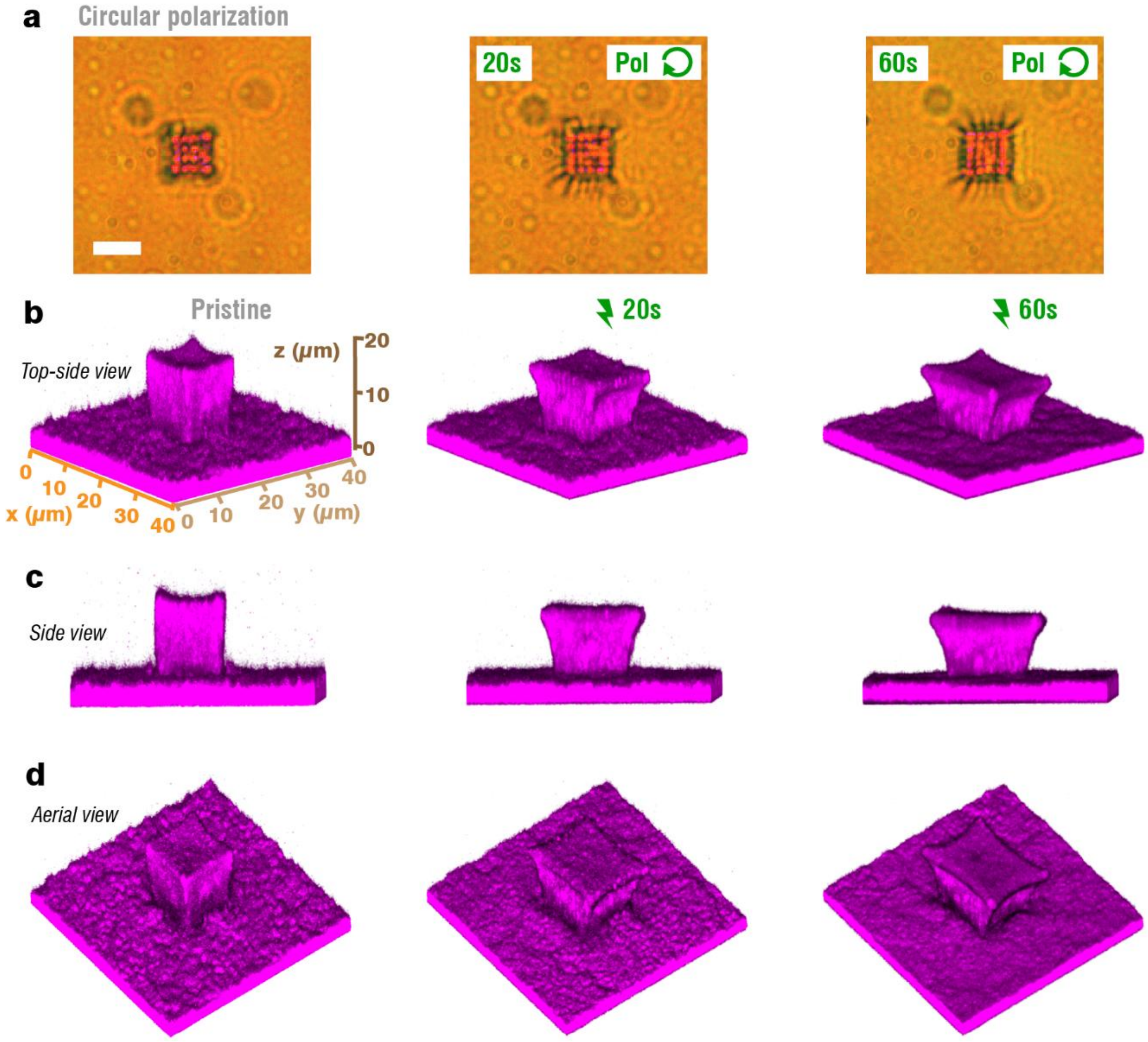

Figure S4. Deformation of micro-actuator cube using circular polarization. a) Optical microscopy images of micro-actuator cubes subjected to circularly polarized illumination (wavelength 532 nm) for increasing illumination times (left to right) at an intensity of 61 Wcm^{-2}. Illumination is incident from the top. Circular polarization stretches the cubes along both in-plane (*xy*) directions. Scale bar (white): 10 μm. b) 3D confocal images of correspondingly illuminated cubes from top-side view. c) Same images from side view. d) Same images from aerial view. The cube assumes a square, mushroom-like shape.

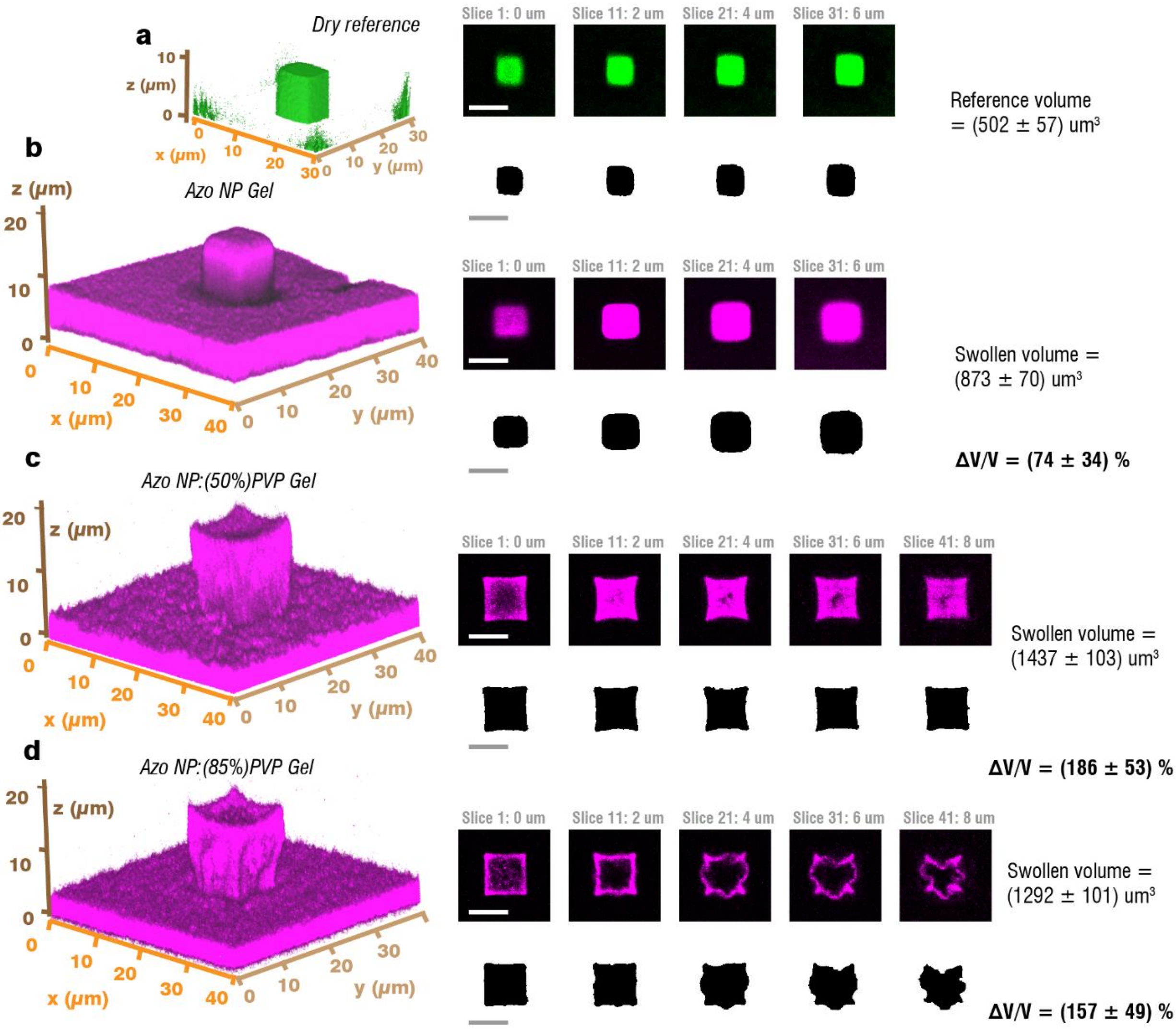

Figure S5. Swelling of different azoGel composite types. a) Confocal images visualizing the three-dimensional shape (left) and cross-sectional slices spaced by 2 µm in the *z*-direction (right) for a dry reference cube. b-d) Same for a swollen Azo NP Gel cube, a swollen Azo NP:(50%)PVP Gel cube and a swollen Azo NP:(85%)PVP Gel cube respectively. Top row images show contrast-adjusted and bottom row images show fully segmented slices for each sample. Scale bars (white/grey): 10 µm. For the dry reference in a, the dry Azo NP Gel cube is imaged using fluorescence (excitation wavelength = 546 nm, emission wavelength = 580 nm), while the swollen cubes are imaged using back-scattered laser light (wavelength = 639 nm), as detailed in the corresponding Methods section. Volumes for each sample, as well as relative volume change of swollen samples with respect to the dry reference are indicated. Error margins are calculated starting from an uncertainty of ± 2 pixels (= 0.31 µm) in lateral dimensions and ± 1 slice (= 0.2 µm) in *z*-direction and computed using uncertainty propagation.

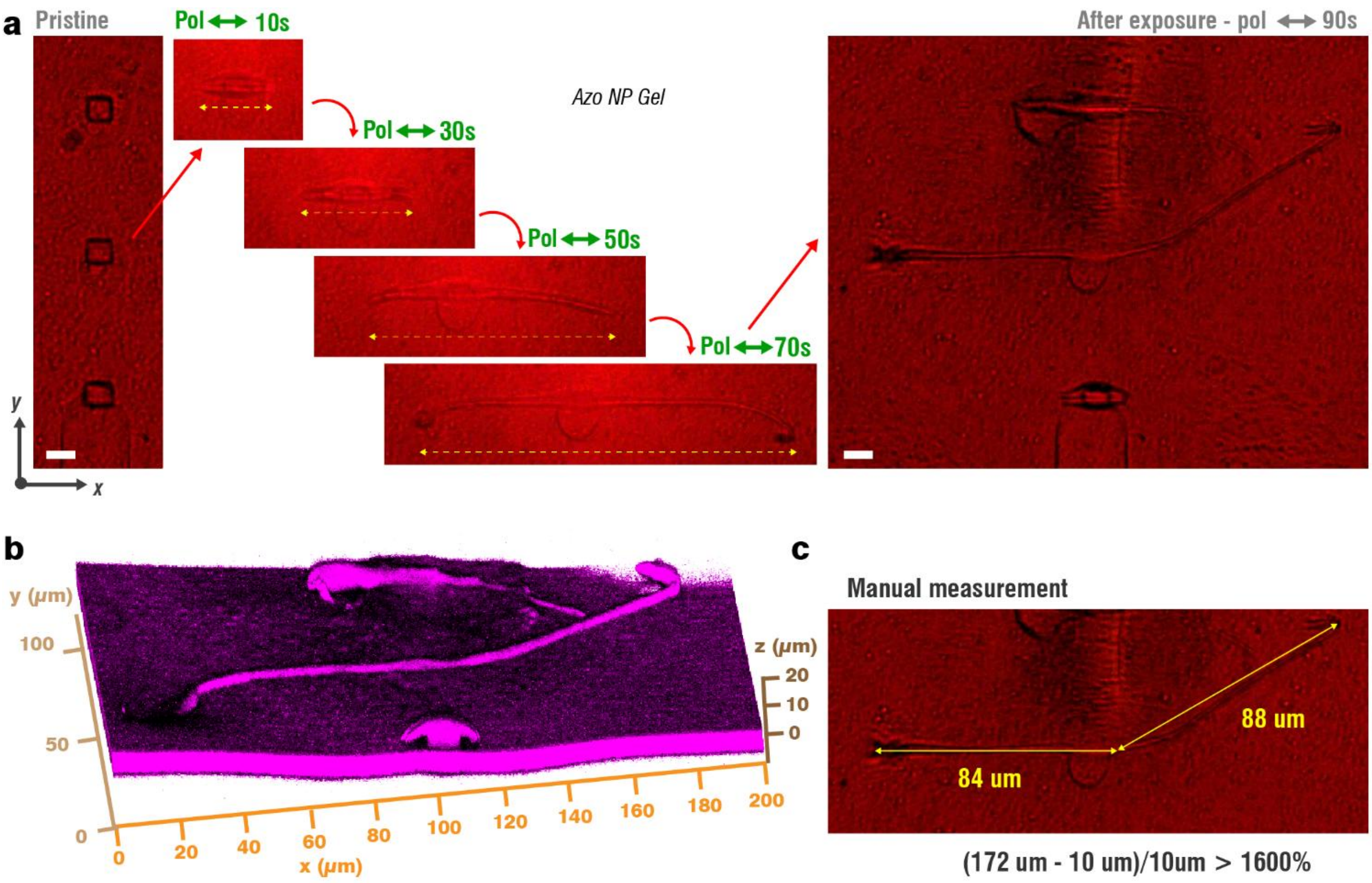

Figure S6. Large deformations of Azo NP Gel at high intensity. a) Continuous deformation of an Azo NP Gel (pure PVA-coated azopolymer particles) cube under top illumination with linearly *x*-polarized green (532 nm) light at an intensity of 126 Wcm^{-2}. During illumination with such high intensity the background becomes considerably brighter. This is due to fluorescence from the azopolymer leaking through the filter which is used to cut out the laser illumination. Scale bars (white): 10 µm. All microscope images have equal scale. b) 3D confocal image showing the heavily deformed, wire-like structure displayed to the right in a, at the end of the illumination interval. c) Conservative manual estimation of the total elongated length of the linearly deformed actuator. Note that at these high deformation extents, the soft structure tends to buckle and float (see also Supplementary Movie S2), making application of the automatic deformation algorithm used elsewhere in the work difficult. Still, a total elongational relative deformation exceeding 1600% can be measured.

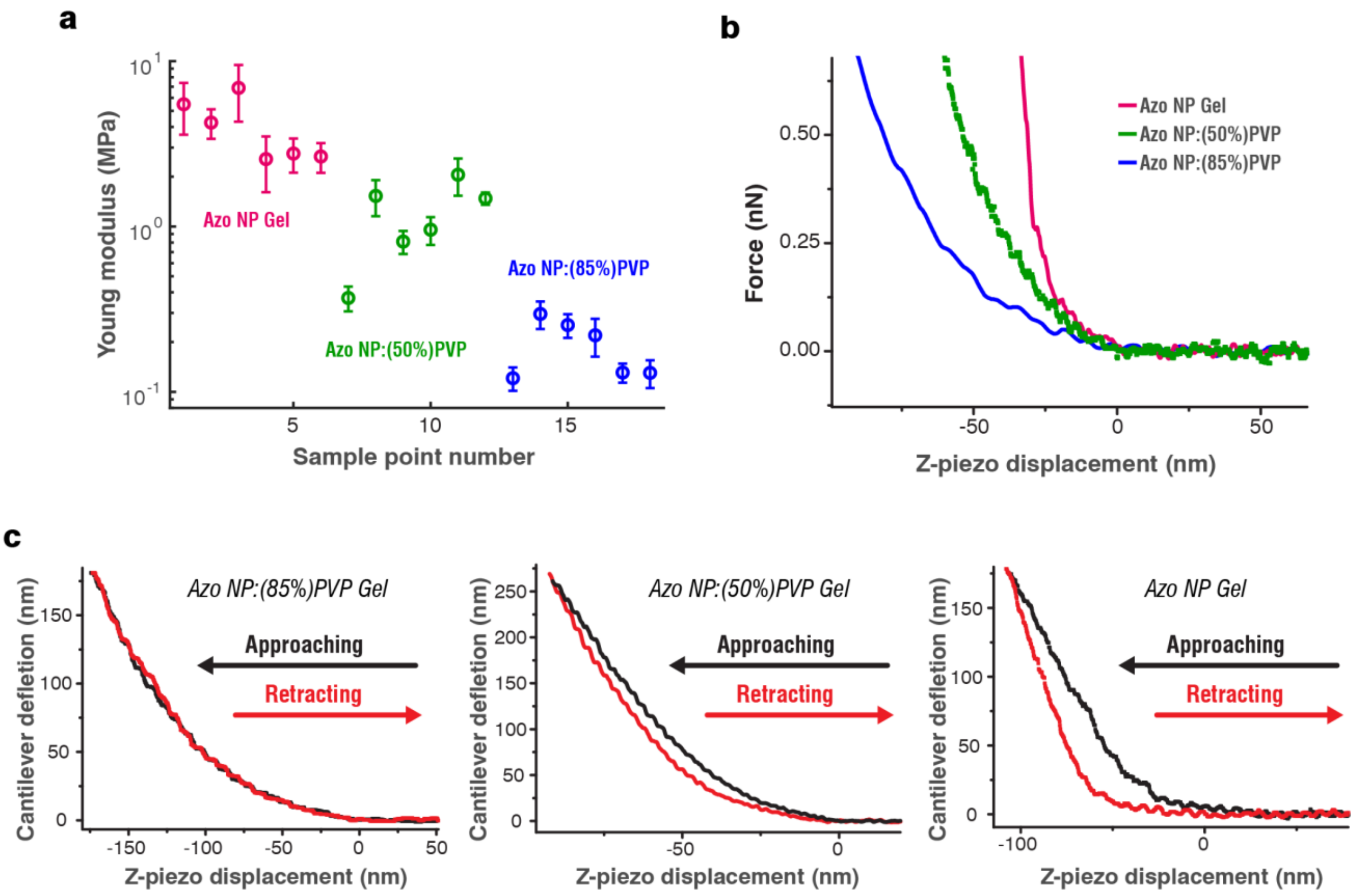

Figure S7. Young Modulus measurements. a) Young modulus measured at different sampling points for the three different types of azoGels. Error bars refer to the standard deviation of measurements in each single sampling point. Note that the composites are probed as a whole, not adjusting for the altered density of rigid azopolymer particles when adding PVP. b) Representative force curves obtained from the different samples. Only the approaching part of the entire force curves, which is the one exploited in the fitting procedure for obtaining the Young's modulus, is reported, and the curves have been aligned to the estimated contact point. c) Approaching/retracting curves show an increasing hysteresis behavior for decreasing PVP network content in the composite gels. Hysteretic behavior is represented by the separation between the approaching and retracting portions of the curves, which is related to a time-dependent behavior of the induced deformation. All the curves have been acquired with the same vertical scanning speed of 2 µm/s.

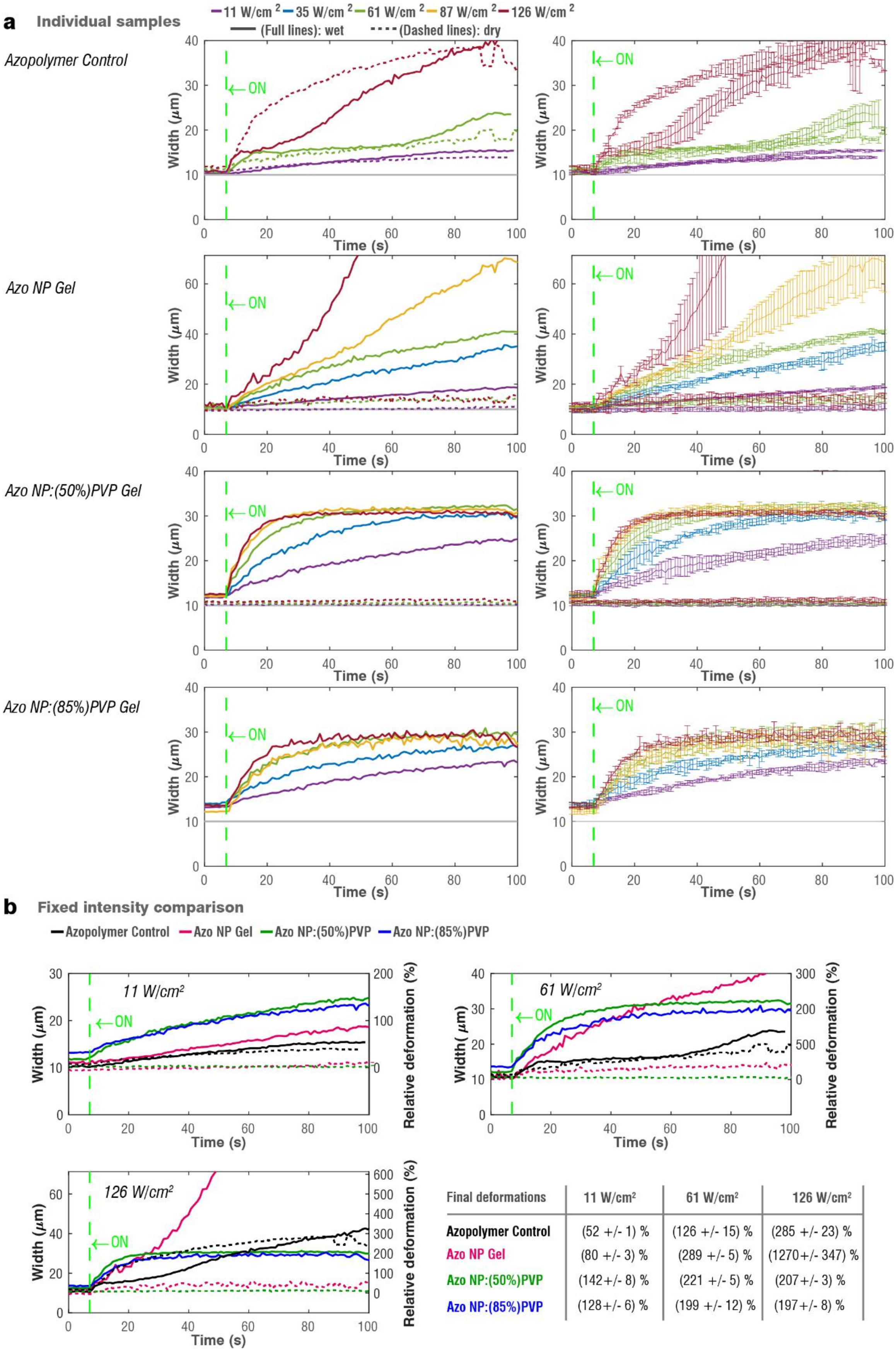

Final deformations	11 W/cm²	61 W/cm²	126 W/cm²
Azopolymer Control	(52 +/- 1) %	(126 +/- 15) %	(285 +/- 23) %
Azo NP Gel	(80 +/- 3) %	(289 +/- 5) %	(1270+/- 347) %
Azo NP:(50%)PVP	(142+/- 8) %	(221 +/- 5) %	(207+/- 3) %
Azo NP:(85%)PVP	(128+/- 6) %	(199 +/- 12) %	(197+/- 8) %

Figure S8. Directional deformation behavior of different azoGel composite types. a) Deformation graphs tracking the average width (from four cubes) vs. time for all considered samples at different illumination intensities. Bare plots (left column) and plots with error bars (right column) referring to the sample standard deviation for the four cubes' deformation values in each case. b) Replotting the graphs from a, grouping the response of the different samples based on illumination intensity. Whilst PVP-

containing cubes (Azo NP:(50%)PVP Gel and Azo NP:(85%)PVP Gel) are the most deforming ones at low intensity (11 Wcm^{-2}), their deformation is limited to values around 200% for any intensity, whereas both pure Azopolymer Control and pure Azopolymer NP Gel can reach far higher deformation as intensity increases. The table on the lower right reports the average relative deformation detected during the last five seconds of illumination in each case, with uncertainty intervals reporting inter-cube sample standard deviation.

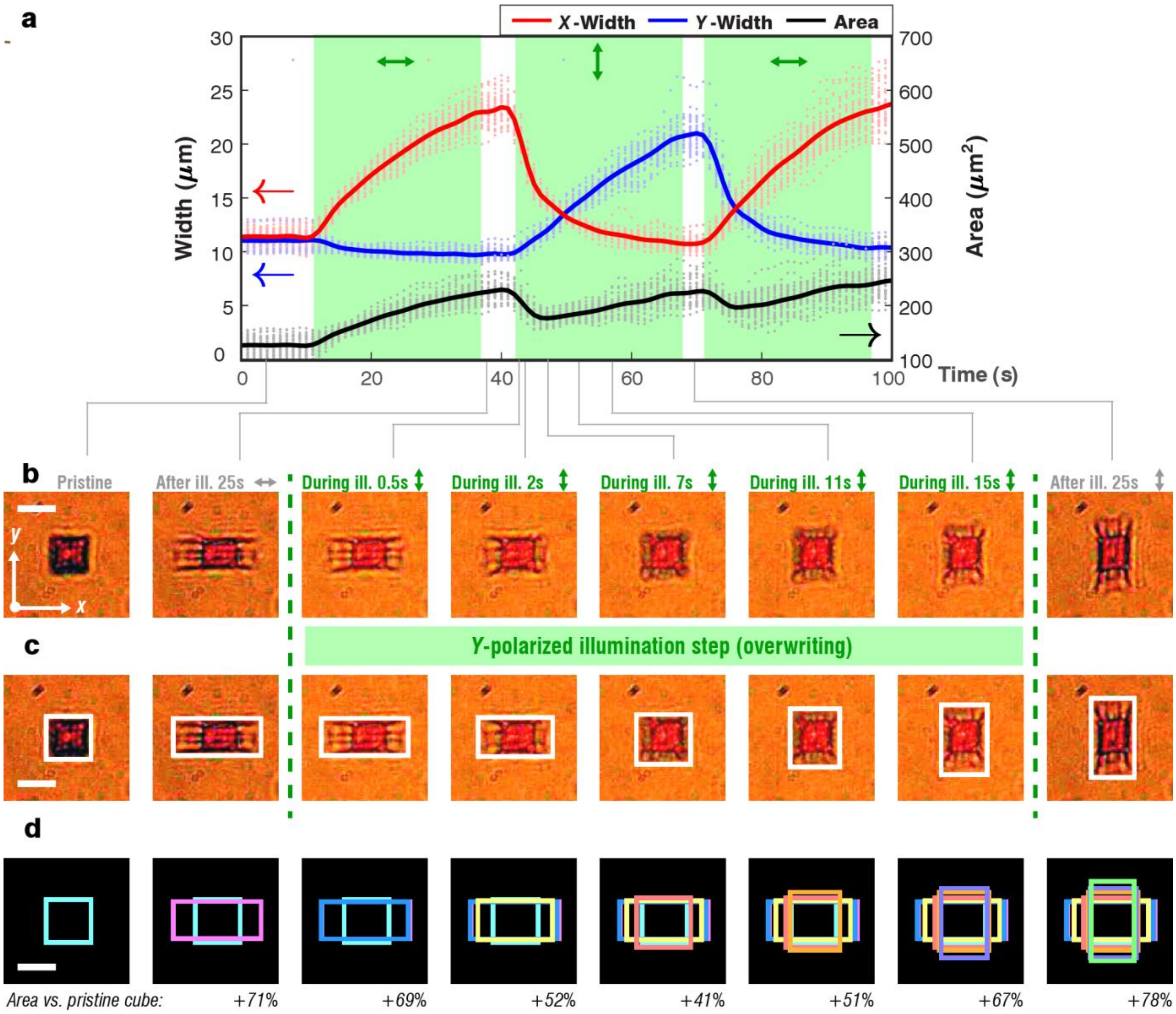

Figure S9. Details of area recovery during overwriting. a) Zoom-in at the beginning of the deformation graph shown in Figure 2c, closing in on the deformation behavior in response to the first three orthogonally polarized illumination steps. b) Successive snapshots of a single cube linked to the corresponding timepoints on the graph by light gray lines (from associated Supplementary Movie S4). The snapshots show (from left to right) the pristine cube, as well as the cube before, during and after the first overwriting step (*y*-oriented polarization). On the successive images, the cube appears to first retract along the previous deformation direction (*x*-axis), before deforming into the new direction (*y*-axis) c) Same snapshots as in b but with overlaid tracking frames. d) Tracking frames only, successively overlaid in different colors, keeping the frames from preceding snapshots for comparison. Percentages below the images show the relative area expansion of the currently tracked state compared to the pristine state for the selected example cube. Scale bars (white): 10 μm.

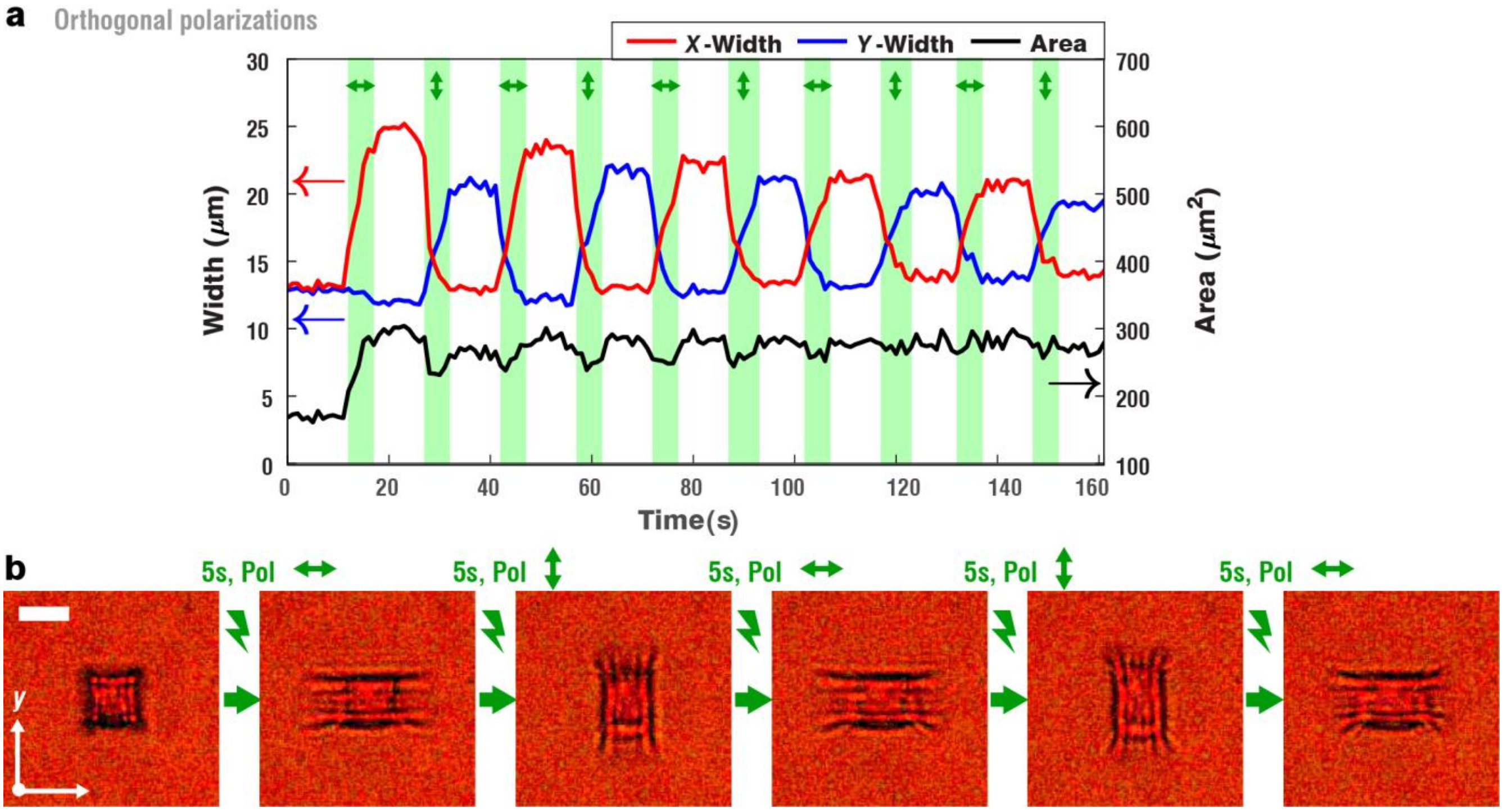

Figure S10. Faster deformation and overwriting using higher intensity. a) Deformation graph tracking the average *x*- and *y*-widths as well as actuator area vs. time for six Azo NP:(50%)PVP cubes, under sequences of illuminations with orthogonal polarizations. Illumination intervals: 5 s, Intensity: 126 Wcm^{-2}, green arrows: polarization direction. Full sequence shown in Supplementary Movie S5. Note that deformation is significantly faster than in Figure 2c/Supplementary Movie S4, due to the roughly four times higher illumination intensity b) Snapshots from Supplementary Movie S5 before/after successive illumination steps. Scale bar (white): 10 μm.

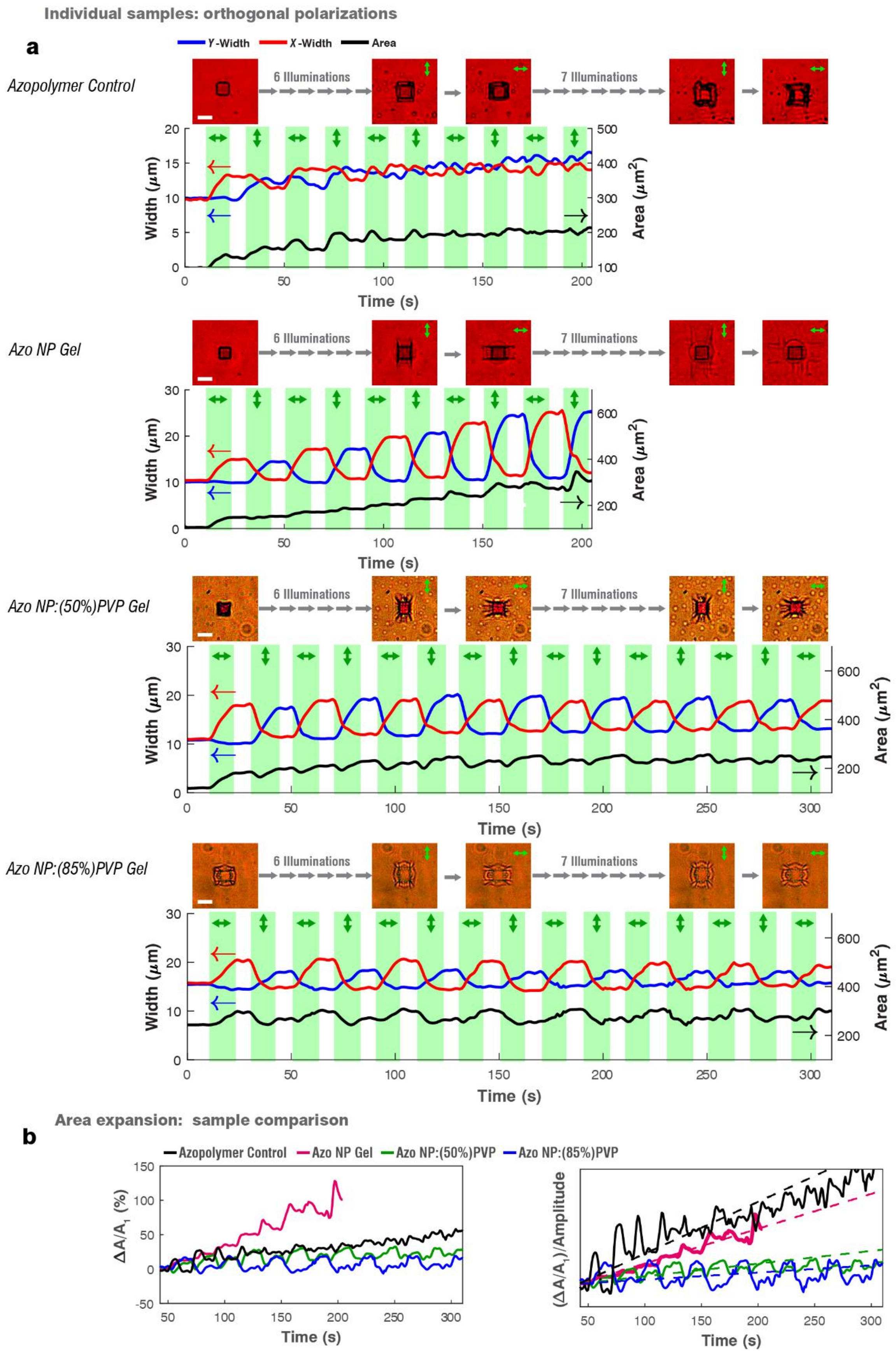

Figure S11. Overwritability assessment for different types of azoGel composites. a) Deformation graphs tracking the average *x*- and *y*-widths as well as actuator area vs. time for all considered samples, under sequences of illuminations with orthogonal polarizations. Illumination intervals: 10 s, Intensity: 39 Wcm^{-2}, green arrows: polarization direction. Full videos shown in Supplementary Movie S6. Snapshots above each graph exemplify the shape of cubes at selected instants along the sequence. Scale bars (white): 10 μm. b) Left: graphs tracking the relative area change of cubes of different azoGel types,

taking the state after the first illumination cycle (one *x*-oriented and one *y*-oriented polarization step completed, labeled as area A_1) as reference. Right: same data but normalizing the relative area change by the average stretching amplitude along the *x*-axis (defined as width after illumination, divided by width before illumination, for *x*-polarized illuminations) displayed by the cubes. This ratio indicates how much of the overwriting amplitude is converted into flattening of the cubes on average (disregarding the first cycle) and is considerably higher for Azopolymer Control and Azo NP Gel samples. Tracking is performed on initial sets of 48 cubes for Azo NP Gel, 16 cubes for Azopolymer Control and Azo NP:(50%)PVP Gel and 8 cubes for Azo NP:(85%)PVP Gel. For Azo NP Gel and Azopolymer Control, the number of successfully tracked cubes decreases down to 15 and 14 respectively during the last cycle, due to residual layer degradation.

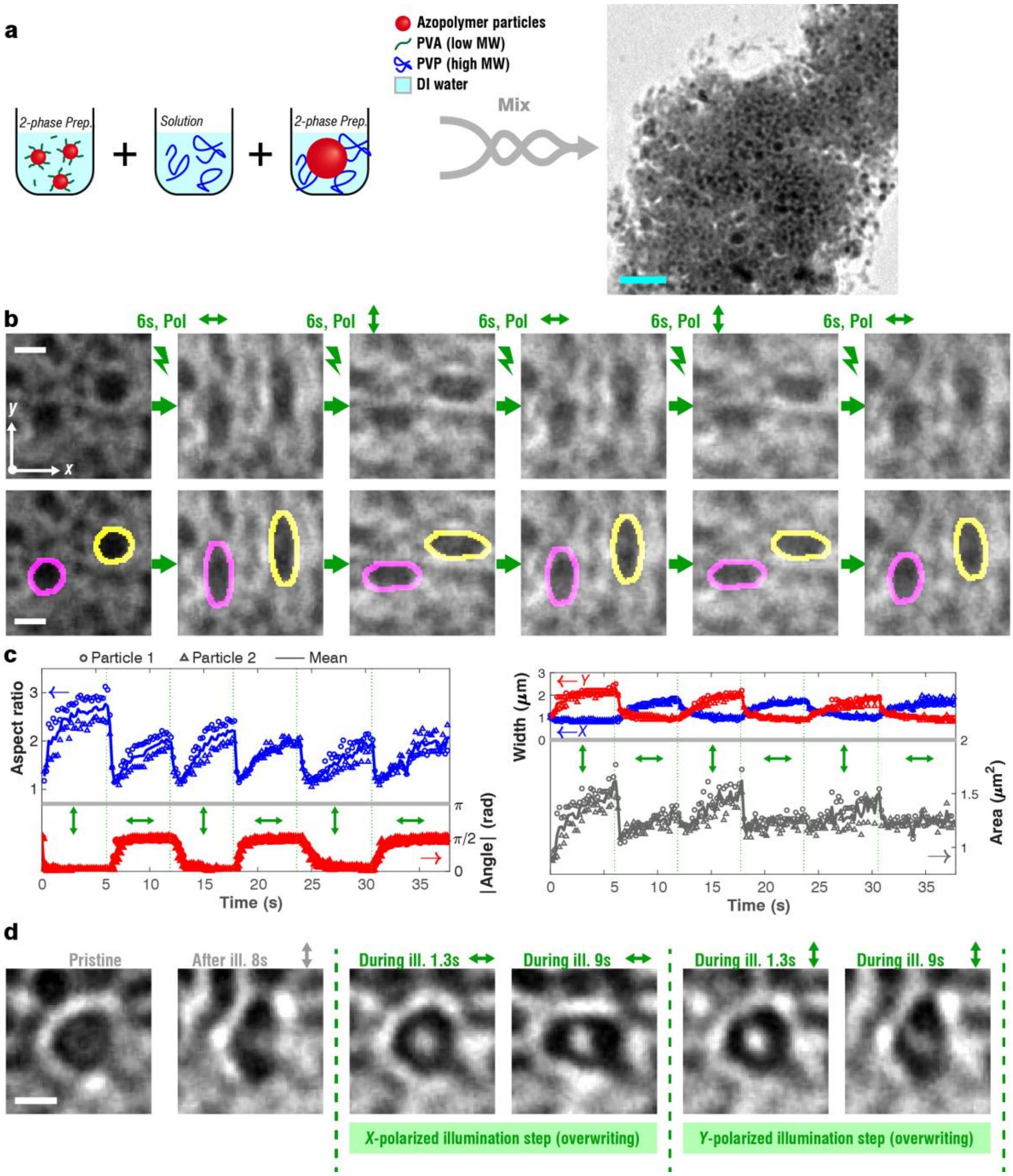

Figure S12. Deformation of embedded large azopolymer particles. a) Modified fabrication protocol for this experiment with addition of a low-density solution of larger azopolymer particles to the Azo NP:(50%) PVP gel. The gel is cast as bulk layer onto a glass substrate with an overview image shown to the right. Scale bar (cyan): 5 µm. b) Snapshots from Supplementary Movie S7 showing two particles (~1 µm in diameter) deformed sequentially with orthogonal polarization steps. Illumination intervals: 6 s, Intensity: 66 Wcm^{-2}. The bottom row shows overlaid ellipses obtained by tracking the particles in ImageJ using the following sequence: *Auto Local Threshold v1.11 ("Phalsankar", 15px, P1 = 0.5, P2 = 0.5)* → *Make Binary* → *Fill Holes* → *Median (3px)* → *Dilate* → *Watershed* → *Analyze Particles.* Occasional split particles (tracking error) were removed manually and the outline replaced by the one from the previous frame. Scale bars (white): 1 µm. c) Graphs showing the aspect ratio, long ellipse orientation angle, widths and area of the two example particles during the whole sequence. In analogy with the cube deformations (see Figure S9), the particles become round and their in-plane area partially recovers during overwriting with orthogonally oriented polarization. d) Snapshots of another large particle shown in Supplementary Movie S7, where the transient round shape, close to the pristine one, appears particularly visible at the beginning of each overwriting step. These observations suggest the presence of restoring forces erasing most of the previous deformation even before the next deformation is inscribed. Illumination intervals: 8 s, Intensity: 118 Wcm^{-2}, green arrows: polarization direction. Scale bar (white): 1 µm.

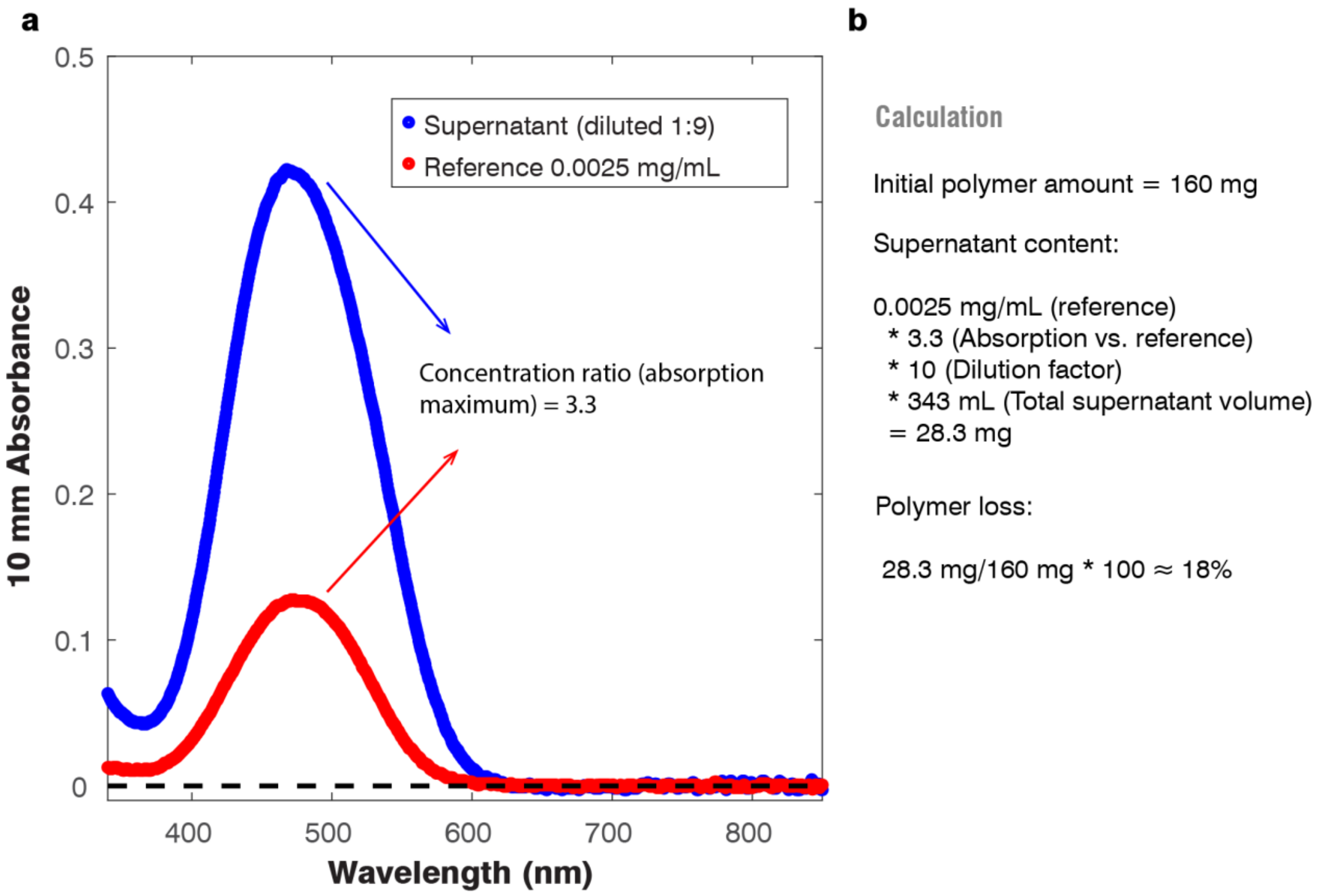

Figure S13. Polymer amount lost during transvasing and centrifugation. The amount of polymer lost before redispersion of the azopolymer particles in the final solution is estimated by combining all the supernatants from centrifugation, as well as leftovers in the particle manufacturing glassware, and comparing against a reference solution of the polymer. a) UV-Vis absorption spectra of the diluted supernatant solution and the 0.0025 mg/mL p(DR1m-co-mma) reference solution under equal path length. The ratio between peaks is used to estimate the total polymer content in the diluted supernatant, neglecting particle scattering contributions. b) Calculation used to obtain the total mass of polymer lost during manufacturing, used to tune the amount of water to add to the final solution such as to obtain a ~30 wt% azopolymer nanoparticle solution.
